# High-ambition climate action in all sectors can achieve a 65% greenhouse gas emissions reduction in the United States by 2035

**DOI:** 10.1038/s44168-024-00145-x

**Published:** 2024-07-24

**Authors:** Alicia Zhao, Kowan T. V. O’Keefe, Matthew Binsted, Haewon McJeon, Adriana Bryant, Claire Squire, Mengqi Zhang, Steven J. Smith, Ryna Cui, Yang Ou, Gokul Iyer, Shannon Kennedy, Nate Hultman

**Affiliations:** 1 Center for Global Sustainability, University of Maryland, College Park, MD USA; 2 Joint Global Change Research Institute, Pacific Northwest National Laboratory, College Park, MD USA; 3 Graduate School of Green Growth & Sustainability, Korea Advanced Institute of Science and Technology, Daejeon, Korea; 4 Global Energy Monitor, Covina, CA USA; 5 College of Environmental Sciences and Engineering, Peking University, Beijing, China; 6 Institute of Carbon Neutrality, Peking University, Beijing, China

**Keywords:** Policy, Climate-change mitigation, Energy modelling

## Abstract

Under the next cycle of target setting under the Paris Agreement, countries will be updating and submitting new nationally determined contributions (NDCs) over the coming year. To this end, there is a growing need for the United States to assess potential pathways toward a new, maximally ambitious 2035 NDC. In this study, we use an integrated assessment model with state-level detail to model existing policies from both federal and non-federal actors, including the Inflation Reduction Act, Bipartisan Infrastructure Law, and key state policies, across all sectors and gases. Additionally, we develop a high-ambition scenario, which includes new and enhanced policies from these actors. We find that existing policies can reduce net greenhouse gas (GHG) emissions by 44% (with a range of 37% to 52%) by 2035, relative to 2005 levels. The high-ambition scenario can deliver net GHG reductions up to 65% (with a range of 59% to 71%) by 2035 under accelerated implementation of federal regulations and investments, as well as state policies such as renewable portfolio standards, EV sales targets, and zero-emission appliance standards. This level of reductions would provide a basis for continued progress toward the country’s 2050 net-zero emissions goal.

## Introduction

The Paris Agreement sets out global goals to reduce greenhouse gas (GHG) emissions in the pursuit of limiting global warming to 1.5 °C above pre-industrial levels. To support this goal, each country has committed to communicating its near-term climate targets through regularly updated nationally determined contributions (NDCs). Many countries have also articulated long-term strategies towards net-zero emissions. Yet, current 2030 NDCs are insufficient for achieving long-term Paris Agreement goals, and therefore ratcheting up and accelerating near-term climate ambition is necessary to bridge this gap^1–3^. Countries are now undertaking the planning and target-setting phase for the next round of NDCs, which are due in 2025. Setting ambitious and achievable 2035 targets will be critical for putting the world on a path toward 1.5 °C.

As the world’s largest economy and second-largest GHG emitter, the United States will be a keystone of global success to deliver sufficiently high levels of ambition. Through its current NDC, the country has pledged to reduce emissions by 50–52% below 2005 levels by 2030, with a long-term strategy to reach net zero by 2050^4,5^. Recent U.S. actions have made substantial contributions toward its climate targets. At the federal level, the historic Inflation Reduction Act (IRA) of 2022 and the Bipartisan Infrastructure Law (BIL) of 2021 are estimated to provide well over $1 trillion for clean energy and carbon storage incentives, transportation infrastructure, methane reduction measures, and more—a collective investment estimated to reduce emissions by 33–40% by 2030^6–10^.

At the same time, U.S. states, cities, and other non-federal actors also play an important role in enhancing climate ambition^11–15^. Sub-national governments in particular have been an important force behind the evolution of increasing U.S. climate action. They have done so through, for example, establishing renewable electricity targets, electric vehicle (EV) sales targets, building codes to increase efficiency and electrification, and more^16–18^. Other non-federal actors, such as businesses and non-governmental organizations, have also supported U.S. climate action through establishing innovative partnerships and collaboration through coalitions (e.g., America Is All In, United States Climate Alliance). Even beyond the significant actions by the federal government, the policy authorities of these subnational governments and other non-federal actors collectively are contributing substantially toward near-term emissions reductions^13–15^.

While combined actions from federal and non-federal actors have brought the United States closer to meeting its 2030 NDC, a gap remains; there is still a 10–17 percentage point difference between the 50–52% reductions needed under the NDC and the estimated 33–40% reductions that would be achieved by on-the-books policies in 2030^6–10^. As the United States begins to consider its next NDC, setting a 2035 target that enables deeper reductions toward net zero in 2050 will be critical. Furthermore, the target should also be rooted in a plausible set of policy actions that can be delivered through both federal and non-federal actions. Existing modeling studies have assessed additional actions needed to achieve the 2030 NDC, including clean electricity standards, accelerated permitting and siting of electricity transmission and renewables, enhanced incentives and sales targets for EVs, stronger building appliance standards that push the market towards all-electric appliances, and more stringent regulations on oil and gas sector methane^7,8,19–21^. However, these policy-driven studies are limited to 2030. Longer-term studies that model deep decarbonization through 2050 have provided insights on broader, sectoral trends needed to achieve net zero^22–25^. These studies tend to model economically optimal pathways through economy-wide carbon price approaches or constraints to achieve sectoral targets rather than detailed policies in each sector. As such, little assessment models a potential 2035 NDC with sector-by-sector actions at the federal and non-federal levels.

In this study, we examine pathways toward an ambitious and plausible 2035 NDC through a suite of specific policies from both federal and non-federal actors across all sectors. We build upon previous work to provide an analysis of options through 2035 that would enable the United States to not only meet its 2030 NDC, but also be on a path toward net zero in 2050^13^. While we aim to push the boundary on maximum ambition in 2035, to ensure the attainability of our policies, we base our modeling assumptions on policy mechanisms that already exist or are being proposed and implement them at different levels of policy ambition for different states.

Our use of an integrated assessment model (IAM) allows us to simulate sectoral interactions. Furthermore, the IAM has resolution for all 50 U.S. states, which allows us to account for specific state-level climate actions. We first quantify the implications of existing actions, including IRA and major climate policies across all sectors and GHGs. Then, we develop a comprehensive suite of new and expanded policy actions, including extended IRA provisions and expanded non-federal climate targets. We find that a comprehensive policy platform of federal, state, and other policies can deliver reductions in net GHGs of 65% below 2005 levels by 2035.

## Results

### Assessing impacts of current policies and additional policies to achieve an ambitious 2035 NDC

In this study, we model federal and state-level policies and measures that have the potential to reduce GHG emissions. We assume that policies are fully implemented and that other non-federal actions are supportive of state-level actions.

We project GHG emissions under two scenarios. The *Current Policies* scenario reflects existing, on-the-books climate actions. Key policy drivers in this scenario include existing state-level renewable portfolio standards (RPS) and zero emission vehicle (ZEV) targets, Corporate Average Fuel Economy (CAFE) standards, IRA tax credits for clean energy, and the IRA methane fee on oil and gas facilities. Table 1 summarizes the modeled policies in both scenarios. A detailed description of these assumptions can be found in Supplementary Notes 6–11.

**Table 1:** Summary of modeled policies under the *Current Policies* and *Enhanced Ambition* scenarios

Sector	*Current Policies* Scenario	*Enhanced Ambition* Scenario
Electricity	Federal actions: • Modeled IRA provisions include: production tax credit (PTC), investment tax credit (ITC) extension, residential clean energy credit, PTC for existing nuclear, energy infrastructure reinvestment financing, and the extension of the 45Q tax credits for captured CO_2_. Non-federal actions: • Current renewable portfolio standards (RPS) • Regional Greenhouse Gas Initiative (RGGI)	Federal actions: • All modeled IRA provisions from the *Current Policies* scenario are extended through 2035 and the 45Q tax credit is enhanced. • The proposed federal standards under Clean Air Act section 111(b) and 111(d) are included. Non-federal actions: • RPS targets are enhanced to at least 75% by 2035 for Tier 1 states, 55% for Tier 2 states, and 20% for Tier 3 states. Federal & non-federal actions: • All unabated coal-fired electricity generation is phased out by 2030.
Transportation	Federal actions: • Modeled IRA provisions include: the clean vehicle credit, alternative refueling property credit, commercial clean vehicle credit, and extension of incentives for biofuels. • BIL funding for light duty vehicle (LDV) and freight truck EV charging infrastructure and funding for school and transit bus electrification are included. • Existing CAFE and GHG emissions standards for LDV and freight trucks are modeled. Non-federal actions: • Major existing incentives for LDV EVs are modeled at the state level. • EV sales mandates for LDVs are modeled to be consistent with California’s Advanced Clean Cars I (ACC I) for five states and consistent with California’s Advanced Clean Cars II (ACC II) for California and 10 other states. • For freight truck electrification, California and 12 other states are assumed to achieve sales targets consistent with California’s Advanced Clean Trucks (ACT) legislation.	Federal actions: • All modeled IRA tax credits are extended through 2035. • Fuel efficiency of LDVs and freight trucks with internal combustion engines are assumed to improve. • A “cash-for-clunkers” style program is assumed to accelerate the retirement of older inefficient vehicles. Non-federal actions: • Tier 1 states are assumed to achieve EV sales consistent with California’s ACC II and ACT legislation, with Tier 2 and Tier 3 states achieving ACC II and ACT sales targets on a delayed schedule. • Vehicle miles traveled reduction policies take place across all states, with annual average per capita reductions ranging from 0.75% to 1.25% between 2025 and 2035. Federal & non-federal actions: • 100% electrification of new bus sales by 2030.
Buildings	Federal actions: • Modeled IRA provisions include: the energy efficient commercial building deduction, energy efficient home improvement credit, energy efficient home credit, home energy efficiency credit, and high efficiency home rebate program. Non-federal actions: • Current state-level energy efficiency resource standards (EERS).	Federal actions: • All modeled IRA provisions from the *Current Policies* scenario are extended through 2035. Non-federal actions: • EERS assumptions are strengthened. • Zero-emissions appliance standards drive space heating and water heating appliance sales to 100% electric by 2030 in Tier 1 states and 2035 in Tier 2 states.
Industry & others	Federal actions: • Modeled IRA provisions include: the 45Q tax credits for captured CO_2_, PTC for clean hydrogen, manufacturing investment tax credit for advanced energy projects, advanced industrial facilities deployment program, and methane emissions reduction program. • Phasedown of HFC emissions consistent with the American Innovation and Manufacturing (AIM) Act. Non-federal actions: No explicitly modeled policies. Federal & non-federal actions: • Implementation of state and federal agriculture and forestry policies, including relevant IRA and BIL provisions, leads to sequestration from land-use, land-use change, and forestry (LULUCF) of −858 MtCO_2_.	Federal actions: • Additional industrial CCS is achieved across cement, biofuels, and pulp and paper production. • No new coal across all industrial sectors. • An economy-wide methane fee. Non-federal actions: • Tier 1 states achieve additional HFC reductions through measures such as the Significant New Alternatives Policy and Refrigerant Management Programs. Federal & non-federal actions: • LULUCF sequestration reaches −926 MtCO_2_ from enhanced actions. • Direct air capture (DAC) removes 31 MtCO_2_ annually by 2035.

*Enhanced Ambition* includes all policies modeled in *Current Policies*.

The high-ambition scenario, called the *Enhanced Ambition* scenario, expands upon *Current Policies* and includes new potential policies from both federal and non-federal actors that are designed to meet the current 2030 NDC and a 2035 NDC that is on the path to net zero. Some of the additional actions modeled include a full phaseout of unabated coal-fired electricity generation by 2030, accelerated ZEV adoption for light-duty vehicle, bus, and freight truck markets, zero-emission appliance standards, a methane fee that covers all sectors, as well as extensions of the existing IRA tax credits. We group the states into three tiers to represent differences in the potential for increased climate ambition. States are tiered based on their current level of climate ambition and historical willingness to lead on climate. Tier 1 states, such as California, Colorado, and New York, follow the most ambitious policies from climate-leading states, such as California’s EV sales targets and zero-emission appliance standards. Tier 2 states, such as Arizona, Michigan, and North Carolina, lag behind Tier 1 states but still increase their current ambition, and Tier 3 states, such as Alabama, Mississippi, and Texas, do not implement major changes. Please see Supplementary Note 2 for more information on state tiering.

**Fig. 1:**
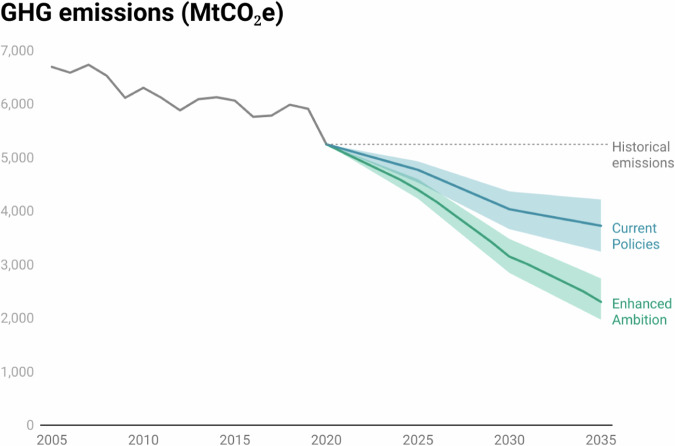
U.S. GHG emissions from 2005–2035. Historical emissions through 2020 are taken from the U.S. Environmental Protection Agency (EPA) inventory^83^. The *Current Policies* scenario achieves 44% emissions reductions below 2005 levels by 2035 in the central case, with a range of 37% and 52%. The *Enhanced Ambition* scenario achieves 65% reductions in the central case, with a range of 59% and 71%.

The *Current Policies* scenario delivers a 40% reduction in GHG emissions in 2030 and a 44% reduction in 2035, relative to 2005 levels (Fig. 1). This is in line with projections from other studies (Supplementary Note 14). Between 2020 and 2030, the average rate of emissions reduction is 123 MtCO_2_e/year. The rate between 2030 and 2035 is around half that at 60 MtCO_2_e/year, as existing policies begin to expire or impacts otherwise taper off.

With expanded and new actions from both the federal government and non-federal actors, the *Enhanced Ambition* scenario delivers 53% (52.6%) in emissions reductions by 2030, at the upper end of the current NDC range. By 2035, reductions reach 65% (Fig. 1). The average rate of emissions reduction from 2020 to 2030 is 208 MtCO_2_e/year, and 167 MtCO_2_e/year between 2030 and 2035. To better capture uncertainties around some technical and economic factors, we also varied assumptions about future GDP and population pathways, technological change, fossil fuel prices, and the size of the land sink in each scenario. These sensitivities suggest that the *Current Policies* reductions could range from 37% to 52% by 2035, while the *Enhanced Ambition* reductions could be as low as 59% or as high as 71% (see “Methods” for further discussion).

While a linear pathway from 50% reductions in 2030 to net zero in 2050 would require an average reduction of 2.5% per year from 2005 emissions levels (achieving 62.5% GHG reduction in 2035), faster rates of decarbonization in the near-term may be required to offset slower reductions later. It is broadly agreed that as economies close in on net zero, decarbonization becomes more challenging due to the remaining emissions from hard-to-decarbonize sectors, including high-temperature heat applications in industry, aviation, shipping^25–27^, and non-CO_2_ emissions from agriculture^28–31^. Our *Enhanced Ambition* suite of policies results in reductions of 3.0% per year (relative to 2005) from 2030 to 2035, demonstrating that deeper reductions are possible through the first half of next decade.

**Fig. 2:**
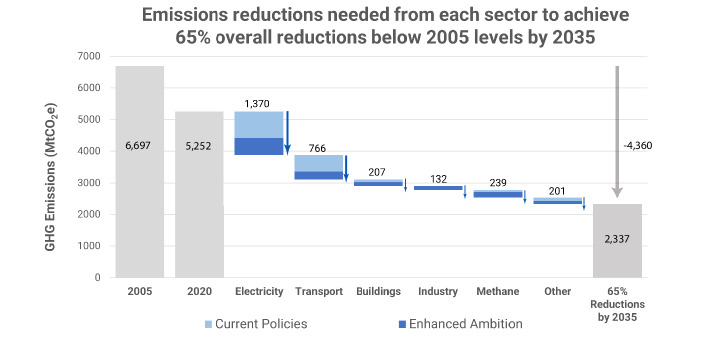
Combined federal and non-federal actions across the U.S. economy allow the United States to achieve a 65% GHG emissions reduction by 2035. Bars show GHG emissions in CO_2_ equivalents, with reductions across the power, transport, buildings, industry, methane, and other sectors under *Current Policies* and *Enhanced Ambition* scenarios. Gray bars represent total emissions in 2005, 2020, and 2035. Blue bars represent emissions reductions needed from each sector between 2020 and 2035, with reductions from the *Current Policies* scenario in light blue, and the additional reductions from the *Enhanced Ambition* scenario in dark blue.

Figure 2 shows the breakdown of sectoral emissions reductions needed to achieve the 2035 reductions in *Enhanced Ambition*, starting from 2020 emissions levels. While U.S. NDCs are typically formulated based on 2005 emissions levels, we assess sectoral reductions and other metric relative to 2020 levels (the last historical year in our model) to provide better context on the magnitude of change needed between today and 2035. The electricity sector has the largest emissions reductions at 1370 MtCO_2_e between 2020 and 2035, contributing to 47% of the overall reductions in this period. The transportation sector contributes to 26% of the overall reductions at 766 MtCO_2_e. Buildings, industry and methane sectors contribute to 20% of the overall reductions at 207 MtCO_2_e, 132 MtCO_2_e, and 239 MtCO_2_e respectively, while the “other” sector, which includes direct air capture (DAC), land-use, land-use change, and forestry (LULUCF) CO_2_, other CO_2_, nitrous oxide (N_2_O), and fluorinated gases, contributes the remaining 7%.

Importantly, we find that the *Enhanced Ambition* scenario would expand mitigation across all sectors and greenhouse gases, with emphasis in sectors like buildings, industry and methane, which are not focal points of emissions reductions in the *Current Policies* scenario. Also, continued action in the electricity and transportation sectors would be needed as these continue to deliver the bulk of overall reductions (Fig. 2).

### Decarbonizing the electricity grid

Notably, the electricity sector delivers the largest emissions reductions in 2035 in both scenarios. Under *Enhanced Ambition*, regulations on coal- and gas-fired power plants and coal phaseout policies reduce electricity generation from fossil fuel sources, while extended IRA tax credits and enhanced state-level renewable electricity targets drive up the share from clean technologies. These combined measures achieve a 76% emissions reduction from 2020 levels by 2030 (1105 MtCO_2_e), which further declines to 94% by 2035 (1370 MtCO_2_e) (Fig. 2). In *Current Policies*, the electricity sector achieves a 54% emission reduction by 2030 (786 MtCO_2_e). However, little progress is made between 2030 and 2035 as IRA tax credits expire and no additional enhancement of renewable electricity targets and power plant regulations is assumed.

**Fig. 3:**
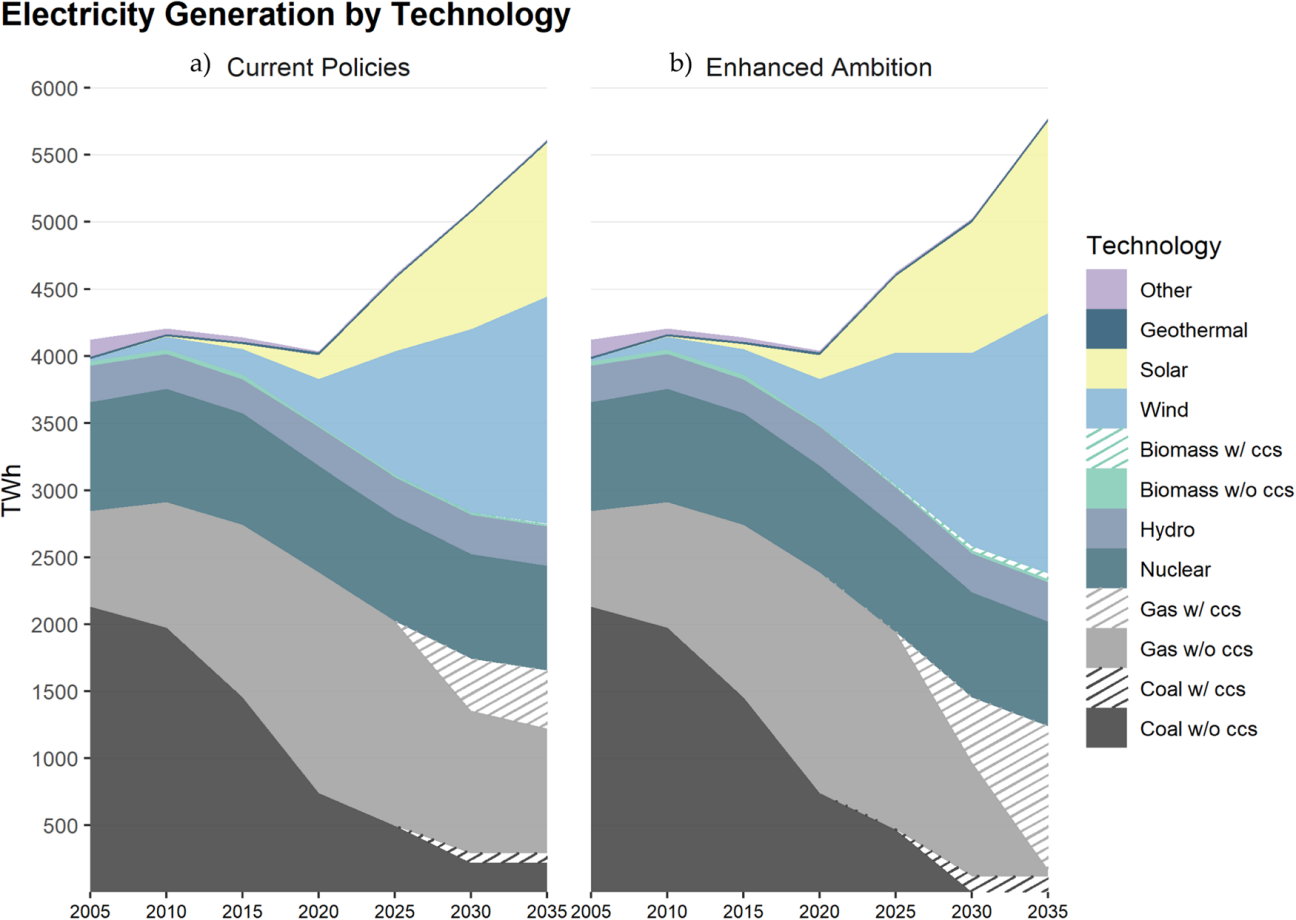
Projected U.S. electricity generation through 2035. Electricity generation is shown in units of terawatt hours (TWh) under (**a**) *Current Policies* and (**b**) *Enhanced Ambition*. By 2035, electricity generation reaches 50% renewable and 78% clean under the *Current Policies* scenario, and 57% renewable and 96% clean under the *Enhanced Ambition* scenario. Renewable sources include solar and wind technologies. Clean sources include solar, wind, geothermal, nuclear, hydropower, and biomass and CCS technologies.

Total electricity demand increases by 41% from 3739 TWh in 2020 to 5301 TWh by 2035 under *Enhanced Ambition*, compared to 5153 TWh by 2035 under *Current Policies* (Fig. 3). There is not a dramatic difference in electricity demand between scenarios despite different levels of end-use electrification due to the additional efficiency measures implemented in *Enhanced Ambition*. Nevertheless, these electricity demand projections require an acceleration from historical trends up to 2023, which is addressed in the Discussion section (Supplementary Fig. 12).

Under *Enhanced Ambition*, solar and wind generation increase by seven fold and four fold from 2020 to 2035, respectively, with wind making up a slight majority of renewable generation. From 2020 levels, solar generation increases by 803 TWh in 2030 and 1273 TWh in 2035; wind generation increases by 1098 TWh in 2030 and 1625 TWh in 2035. For comparison, the highest decadal rates of coal and gas expansion are 575 TWh (1978–1998) and 639 TWh (2009–2019), respectively^32^. This level of renewable generation growth is not consistent with historical trends up to 2023 and would require accelerated deployment, as discussed further below (Supplementary Fig. 11).

All unabated coal-fired power plants are retired by 2030, which is consistent with historical trends up to 2023 (Supplementary Fig. 10). Generation from gas with CCS begins in 2025 at less than 1% of the generation mix, but rises to 18% by 2035 as it replaces unabated gas generation. Only peaker gas plants and gas back-up generators remain in 2035 to stabilize the power grid. Correspondingly, *Enhanced Ambition* achieves a generation mix that is 96% powered by clean technologies (Fig. 3). Clean sources include solar, wind, geothermal, nuclear, hydropower, biomass, and CCS technologies.

### Rapid electrification of passenger and freight vehicles

The transportation sector is the second largest source of emissions reductions in both scenarios, with electrification of road vehicles being the primary driver of these reductions. Transportation emissions under *Enhanced Ambition* decline by 48% below 2020 levels in 2035 (766 MtCO_2_e), compared to 33% under *Current Policies* (519 MtCO_2_e) (Fig. 2).

**Fig. 4:**
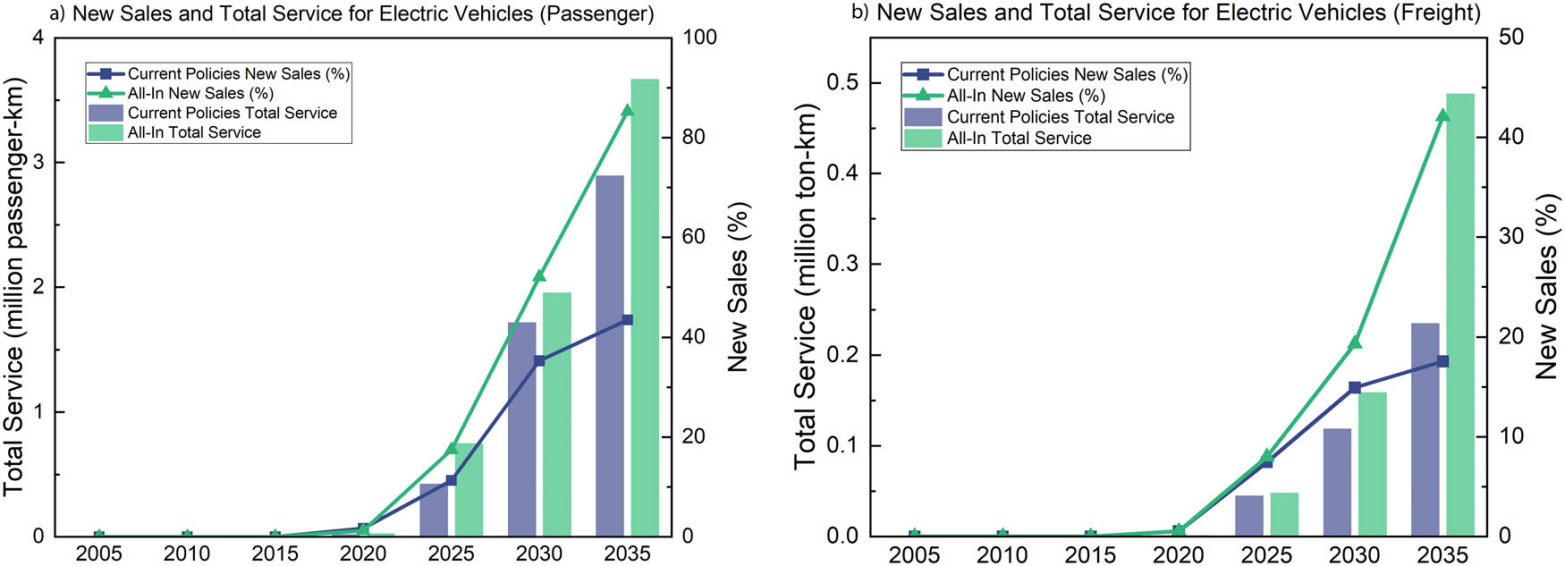
Projected U.S. EV penetration through 2035. New sales and total service are displayed for (**a**) passenger and (**b**) freight EVs. The bars show total EV service in units of million passenger-km for passenger vehicles and million ton-km for freight vehicles, and the lines represent new EV sales. By 2035, EV sales for passenger cars and SUVs increase to 45% under *Current Policies* and 83% under *Enhanced Ambition*. EV sales for freight trucks increase to 18% under *Current Policies* and 42% under *Enhanced Ambition*. Note that EV sales under *Current Policies* flatten between 2030 and 2035 as a result of IRA tax credits expiring before 2035.

Extended IRA tax credits for EVs, strengthened CAFE standards, and enhanced state-level EV sales mandates help accelerate road transport electrification in *Enhanced Ambition*. Electric light-duty vehicle (LDV) sales come within a few percentage points of the current administration’s target of 50% EV sales by 2030, and reach 83% by 2035, in contrast to 45% by 2035 under *Current Policies* (Fig. 4). LDV EV sales projections are consistent with historical trends up to 2023, assuming continued growth (Supplementary Fig. 13). Freight trucks reach 42% EV sales by 2035 under *Enhanced Ambition*, which is more than double the sales under *Current Policies* (Fig. 4).

**Fig. 5:**
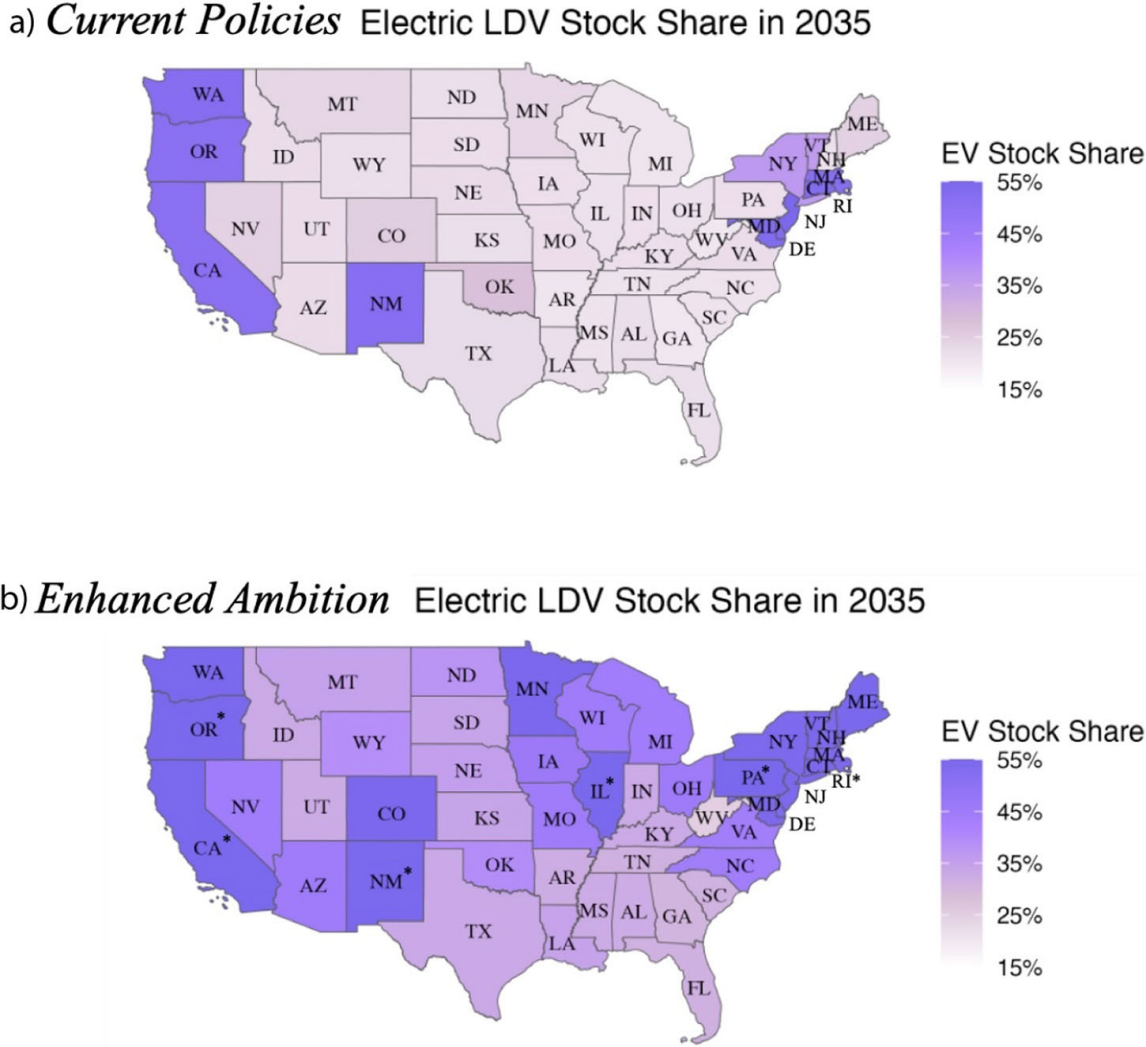
Projected U.S. electric LDV stock across states in 2035. State-level share of electric LDV stock are shown under (**a**) *Current Policies* and (**b**) *Enhanced Ambition* in 2035. States with an asterisk achieve 55% EV stock share or higher (with an upper end of 57%).

However, electric sales will take time to penetrate the existing fleet. Under *Enhanced Ambition*, national LDV stock is only 42% electrified by 2035, which is about half the share of EV sales in that year. Across states, the electric share of LDV stock ranges from 25% to 57% in 2035, with the majority of states achieving 40% or higher (Fig. 5). Under *Current Policies*, the range is 18% to 54%, though most of the states achieve less than 25% (Fig. 5).

Beyond electrification, reducing travel demand is another key lever to help meet decarbonization goals^33^. *Enhanced Ambition* implements state-level policies that reduce vehicle miles traveled (VMT). VMT reduction policies aim to reduce the use of single-occupancy vehicles and promote sustainable modes of transportation (e.g., expansion and enhancement of transit and active transportation options, zoning changes and infill incentives, commuter benefit programs, congestion pricing). With the incorporation of these policies, road passenger service is assumed to increase only by 9% from 2020 levels by 2035 under *Enhanced Ambition*, compared to 28% under *Current Policies*.

### The role of the building and industry sectors

**Fig. 6:**
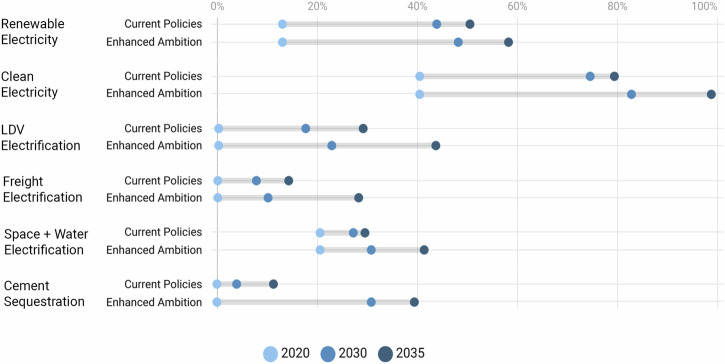
Key metrics, in percentage terms, are compared across *Current Policies* and *Enhanced Ambition*, showing the change between 2020, 2030 and 2035. Renewable electricity is measured by the share of electricity generation from solar and wind technologies. Clean electricity is measured by the share of electricity generation from solar, wind, geothermal, biomass, nuclear, hydro, and CCS technologies. LDV electrification is measured by the share of electricity in passenger car transport service. Freight electrification is measured by the share of electricity in freight truck transport service. Space and water heating electrification is measured by the share of electricity in total final energy from commercial and residential heating. Cement sequestration is measured by the percentage of cement sector process emissions that are captured by cement CCS.

In the buildings sector, extended IRA electrification and efficiency incentives pair with state-level zero-emission appliance standards and enhanced efficiency standards to drive emissions reductions of 38% between 2020 and 2035 (207 MtCO_2_e) under *Enhanced Ambition*, compared to 16% (85 MtCO_2_e) under *Current Policies* (Fig. 2). Most of the electrification incentives and standards target hot water and space heating appliances: the electric share for these appliances increases from 21% in 2020 to 40% by 2035, compared to 29% under *Current Policies* (Fig. 6).

The industrial sector has fewer near-term decarbonization opportunities compared to the other sectors, though it is still an important part of reaching net zero. *Enhanced Ambition* delivers a 12% emissions reduction between 2020 and 2035 (132 MtCO2e), compared to a growth of 4% (43 MtCO2e) under *Current Policies* (Fig. 2). In both scenarios, IRA investments increase electrification and accelerate the retirement of older, inefficient fossil fuels, with electricity shares of around 20% by 2035. However, *Enhanced Ambition* assumes additional ambition unlocked by IRA investments: no new coal across all industrial sectors, as well as high-ambition industrial carbon capture policies. *Enhanced Ambition* achieves 77 MtCO_2_ sequestered from cement, ethanol, and paper pulp CCS technologies, compared to 5 MtCO_2_ of sequestration from cement and ethanol CCS technologies under *Current Policies* (Fig. 6).

### Setting the stage for 2050 with methane abatement, carbon removal technologies, and land sector policies

Comprehensive methane mitigation can significantly slow near-term warming and ease the burden on CO_2_ to achieve global climate targets^28,29^. With a methane fee that covers all sources—including agriculture and waste— to incentivize emission reduction measures, *Enhanced Ambition*’s methane emissions decrease by 30% (239 MtCO_2_e) between 2020 and 2035, in contrast to 5% (41 MtCO_2_e) under *Current Policies*.

Negative emissions through both natural and technological carbon removal will be increasingly important for meeting net zero as they help to balance the hard-to-abate emissions sources^34^. Historically, the LULUCF sink has remained stable or growing, but it can shrink in the future without effective climate policies and due to the impacts of climate change itself^35,36^. As a result of more ambitious lands sector policies under *Enhanced Ambition*, LULUCF sequestration increases by 73 MtCO_2_ from 2020 to 2035, resulting in a sink of −926 MtCO_2_ in 2035. In contrast, LULUCF sequestration increases by only 29 MtCO_2_ under *Current Policies*, reaching a sink of −882 MtCO_2_ in 2035.

Additionally, two carbon dioxide removal (CDR) technologies are explicitly represented in this study. Direct air capture (DAC) of CO_2_ is introduced after 2030 only in the *Enhanced Ambition* scenario, sequestering 31 MtCO_2_ annually from the atmosphere by 2035. Biomass energy with carbon capture and storage (BECCS) in the power and industry sectors also plays a role in the *Enhanced Ambition* scenario, sequestering over 100 MtCO_2_ annually in 2035, compared to 4 MtCO_2_ under *Current Policies*.

## Discussion

The period of 2030 to 2035 represents a transition period in the path to net zero in the United States. Broadly, the bulk of reductions in the 2020’s are expected to come from continued deployment of low-cost renewables in the power sector, combined with increasing levels of EV deployment in the transportation sector. This analysis shows that these trends continue in the period from 2030 to 2035, with rapid and large decreases in the power and transportation sectors. However, there is a critical change from previous NDCs: high-ambition pathways will require the addition of substantial reductions across other sectors of the economy. Electrification and efficiency improvements in the buildings and industry sectors, sequestration from CCS technologies, additional methane mitigation—particularly for fossil fuels and agriculture—and land sink enhancement will all be part of achieving a pathway on track to net zero. High levels of reductions in 2035 will therefore require acceleration of policy actions in these areas in the near term.

Our results show that the transportation sector has the largest opportunity for electrification in the near term. However, beyond instating ambitious sales requirements for EVs, achieving the modeled levels of electrification will require large investments in EV charging infrastructure, including incentives for property owners and businesses as well as specific funding for low-income and disadvantaged communities, which has been a focus in California^37^. Scaling the electrification of freight trucks will be especially challenging given the limited supply at present, the charging needs across state borders, and high associated costs, especially for long-haul trucks^38^. Additionally, some uncertainties remain about the capacity of vehicle supply chains to meet sales targets, as many materials necessary for EV production have been classified as critical or near-critical supply risks, such as lithium, cobalt, nickel, and platinum^39^. Increased demand for critical minerals underscores the necessity of using materials efficiently and addressing ethical sourcing concerns. Due to international sourcing, the critical minerals supply chain is sensitive to disruptions like natural disasters and political turbulence^40^. Furthermore, the extraction process itself is associated with substantial environmental justice concerns^41^. Responsible supply chain management initiatives can help mitigate these risks, for example the framework for assessing supply chain due diligence by the OECD, which provides detailed recommendations for companies to avoid contributing to conflict in high-risk areas^40^.

Electrification also poses challenges for the buildings and industrial sectors. Most buildings in the United States were built many decades ago and require more extensive and expensive retrofits than new buildings^42,43^. Heat pumps for space and water heating have higher capital costs for consumers, though operational costs can vary^42^. The expansion of electrification is further challenged by building codes, state and local bans on fossil fuel bans, opposition from gas companies, and consumer perception^42–44^. Despite these barriers, heat pumps outsold gas furnaces for the first time in the United States in 2023, spurred partially by the IRA tax incentives^45^. Additional incentives to make electric appliances cost effective coupled with education and outreach initiatives could further promote electrification in this sector. The industrial sector faces similar barriers, plus the extreme temperatures needed for high-heat applications, such as cement, have limited technical options and drive up the cost of electrification^42,46,47^.

With successful electrification comes a parallel need for the rapid expansion of low-carbon electricity generation. In China, emissions from energy combustion increased in 2023 despite large additions of renewables and EVs, as the growth in clean energy was insufficient to keep pace with electricity demand^48^. A similar phenomenon has recently emerged in the United States where utilities have announced plans to build new gas plants in response to a surge in power use^49^. This is a concern especially as renewable generation growth is not currently on track to the levels needed by 2035 (Supplementary Fig. 11). Over 1 TW of clean generation capacity is stuck in the interconnection queue^50^. Updating permitting processes and expanding transmission systems will be key to support modeled levels of clean energy generation. This can include streamlining siting and permitting processes for clean energy projects, completing environmental impact statements within a specified time frame, and soliciting early input from relevant stakeholders, as the state of Washington has mandated^51^. Engagement with communities can minimize the possibility of local opposition, which has resulted in additional project delays and cancellations^52,53^. The lack of high-capacity, long-distance transmission also contributes to the interconnection backlogs^50,54^.

In addition, demand-side management can curtail electricity demand growth and support the integration of increasing levels of variable generation^55^. *Enhanced Ambition* includes energy efficiency measures and VMT reduction policies, which taper overall demand, but also important are demand-response programs that can shift the electric usage during peak hours to minimize impact on the grid. Flexible vehicle charging, for example, could span multiple days and therefore aid in reducing demand during critical events^56^. Existing incentives within the current regulatory system for utilities are a major barrier toward achieving clean energy targets. Investor-owned utilities profit by maximizing energy sales—they are therefore disincentivized to promote measures that would reduce electricity demand. Additionally, the conventional utility business model has focused on affordability and reliability, which can conflict with investments in renewable energy, energy efficiency, and new technologies. Utility reform is thus needed to realign these incentives with climate goals^57^.

Emerging technologies can help maintain grid reliability and abate emissions in hard-to-decarbonize sectors^22–25^. Yet the rapid and deep diffusion of new technologies is challenging, owing to factors such as public perception and opposition, high costs compared to incumbent technologies, slow market response, and lack of necessary institutional structures^58^. For CCS technologies, there is some indication that the modeled levels of deployment can be achieved. The United States has robust policies and investments for all stages of CCS development, large geological storage resources, and a growing number of operational, large-scale CCS facilities.^59,60^ Still, higher rates of project realization are needed^61^.

Deployment of CDR technologies will almost certainly be required to offset residual emissions to reach net zero by 2050 in the United States and to limit the amount of global temperature overshoot above 1.5 °C^1,62^. Unrealistic estimates of CDR levels, however, can risk inadequate near-term mitigation and lead to large temperature overshoots^63^. Our *Enhanced Ambition* scenario deploys non-trivial amounts of CO2 removals from DAC and BECCS, which will require significant scale-up from today’s levels, though our numbers are conservative compared to estimates from other studies^64–66^. DAC is one of the most expensive forms of CO_2_ removal, and as such, successful scale up will depend on reductions in cost, regulatory mechanisms and incentives, and maturation of the carbon removal marketplace^65^. It will be important to ensure that any renewable electricity used to power DAC is not competing with grid decarbonization^65^. BECCS also has its challenges, including the costs associated with potentially transporting biomass and CO_2_ over long distances^66^. Accelerating demonstration and pilot projects across different environments in the next few years will be important to achieve cost reductions and make emerging technologies more economically viable for large-scale deployment^67^.

Our policy platform is designed to require maximum ambition from federal and non-federal actors within the context of the current climate policy landscape. However, we are not able to capture different political, social, and behavioral factors, which can create uncertainties around uptake of our modeled policies. For example, governance structures, lack of public support, and industry and interest group opposition could hinder efforts to enact robust climate policies^68^. Policy implementation could provide additional setbacks. For instance, while we model the IRA tax credits at full implementation, the effectiveness of some provisions depend on uptake by state, local and individual actors, and some policies could be watered down upon finalization. At the non-federal level, states are behind on adopting additional policies to meet their commitments, and utilities who have pledged to decarbonize are not following through with the corresponding investment decisions^69,70^. Moreover, uncertainties exist around policy durability, with rollbacks of current policies possible in the future. Under past administrations, such rollbacks have included CAFE standards, methane standards for new and old emitters, and maintenance of old standards in lieu of updating and enhancing them^71^. Investment-led policies that require both Executive and Congressional support, like the IRA, are generally more durable than executive orders and regulatory processes. The fact that investments have primarily accrued in conservative and swing states further strengthens the durability of the IRA^72^.

While no strategy can generate absolute certainty, embedding ambitious climate action at multiple levels of government can support a more durable climate strategy and mitigate potential political backsliding if future elections return less climate-friendly governments. Under this framework, other actors can pick up the slack if individual states and cities roll back policies. California’s 100% ZEV requirement, for example, has become embedded within industry through six automakers’ commitment to comply with the sales requirement through 2030^73^. Financial incentives at the federal level can also help advance climate action in states where additional regulations are unlikely; much of the renewable credits from IRA, for example, are being used to build out clean energy in states with no RPS^74^. In contrast, relying on a single law like a national carbon price would make the United States more vulnerable to different setbacks and changing administrations.

The model used for our research, GCAM-USA-CGS, has many features that make it well-suited for this type of analysis. These include its ability to offer full coverage of all emitting sectors across the economy, beyond standard energy sector CO_2_-only studies, as well as its ability to link the U.S. states with regions in the rest of the world. Furthermore, the model includes dynamic feedbacks across sectors, a rich technological representation, and emissions, energy, and trade details for 50 states. Even so, our analysis has limitations. The model has difficulty capturing the uncertainty in building out transmission and distribution infrastructure– which other studies have shown to be critical in power sector decarbonization^21^. Additionally, this study models policies at the state and national levels, and assumes that other non-federal actions (e.g., cities, businesses) would be embedded in these policies. Yet, climate actions from these actors have been shown to achieve reductions beyond current state and national pledges^14,15^. Not explicitly accounting for their actions therefore introduces further uncertainty to our results.

Charting a plausible, high-ambition emissions reduction pathway is the focus of this analysis. Despite limitations, our analysis provides valuable information on the magnitude and variety of actions needed to set a U.S. 2035 NDC target that will be both sufficiently ambitious and, though likely challenging, also plausible to achieve. Furthermore, it highlights the importance of accelerated implementation of policies that currently exist or are under consideration, such as IRA investments, EPA regulations, and state-level renewable portfolio standards, EV sales targets, VMT reduction policies, zero-emission appliance standards, and cement CCS targets. Our comprehensive results can serve as useful reference points for state and other decision makers as they formulate their climate targets. The modeling tool is publicly available and can be used to analyze specific state-level results, with the caveat that modelers need to carefully examine the historical data and assumptions for that state. For example, GCAM-USA has been used to model an ambitious pathway for Maryland^75^.

There is a key role for a broad suite of actors, including federal, state, and local governments, to continue to build new policies that are durable over the long term to ensure that the United States stays on a 1.5 °C-compatible pathway. Moreover, a U.S. commitment to an appropriately ambitious 2035 NDC would also encourage other countries to ratchet up their own ambition in their NDCs, helping curb overall global emissions to keep the goals of the Paris Agreement within reach.

## Methods

### Overview of modeling approach

Policy representation in our modeled scenarios is built upon bottom-up aggregation tools and data analysis to evaluate and quantify the impacts of state-level policies and climate actions in isolation and within specific sectors. We then used this information with policy levers at the national level in GCAM-USA-CGS to estimate the economy-wide implications of these associated policies. The overall modeling approach used was consistent with previous analysis, including Accelerating America’s Pledge (2019)^13^, An All-In Climate Strategy Can Cut U.S. Emissions by 50% by 2030 (2021), Blueprint 2030 (2021), and Beyond 50: An All-In Pathway to 2030 (2023)^7,13,76–78^. GHG emissions in this work are reported in terms of CO_2_ equivalents using IPCC AR5 100-year GWP values, consistent with current (2023) UNFCCC reporting guidance^79^ (Supplementary Note 13). Please see Supplementary Note 4 for more detailed information on our overall modeling approach.

GCAM-USA-CGS is based on the open-source release of GCAM-USA 6.0^80^. GCAM-USA-CGS has been updated for the purposes of this study, for example, to reflect the latest renewable energy costs^81^. It is also calibrated to the latest non-CO_2_ marginal abatement cost curves from the U.S. Environmental Protection Agency^82^. GCAM-USA tracks emissions of a range of GHGs and air pollutants from energy, agriculture, land use, and other systems. The energy system formulation in GCAM-USA consists of detailed representations of depletable primary sources such as coal, gas, oil, and uranium, in addition to renewable resources such as bioenergy, solar, wind, and geothermal. GCAM-USA also includes representations of the processes that transform these resources into final energy carriers, such as oil refining and electric power. These energy carriers, in turn, are used to deliver services to end users in the buildings, transportation, and industrial sectors. The electric power sector includes representations of a range of power generation technologies, including those fueled by fossil fuels, renewables, bioenergy, and nuclear power. Please see Supplementary Note 3 for more detailed information on GCAM-USA-CGS.

All modeled policies in GCAM-USA-CGS are implemented at the state and/or national levels. Policies and actions from city governments, businesses, and institutions are assumed to be embedded within or supportive of the state and/or national level policy representation in the model, and are therefore not explicitly modeled. Regions in the rest of the world are assumed to roughly follow emissions pathways consistent with their announced NDCs.

Model parameters in GCAM-USA-CGS were varied according to information from our bottom-up aggregation analysis or changed directly for policy drivers where bottom-up aggregation was either not feasible or not necessary in the case of small-scale potential impacts. The purpose of this analysis is to assess the national emissions reduction potential in the United States for the policies modeled in our scenarios. Accordingly, non-federal policies and actions are only modeled to the extent that doing so would have a meaningful impact on the national-level emissions outcome. In some policy areas, we did not specify state-level variation in policy implementation—for example, with 100% bus electrification – as assessment of the national-level emissions impact did not require state-level precision. Please see Supplementary Note 5 for more information and federal and state policy interactions, and Supplementary Note 16 for more information on our bottom-up aggregation analysis.

### Modeled scenarios

The *Current Policies* scenario includes existing federal policies – including several climate and energy provisions from IRA and BIL – and non-federal policies. Existing state-level RPS, CAFE standards, and several IRA tax credits for renewable electricity generation, EVs, and CCS are among the key policy drivers in this scenario.

The *Enhanced Ambition* scenario models GHG emissions reductions achievable under a comprehensive climate strategy that builds upon the policy framework in the *Current Policies* scenario with enhanced non-federal and federal action. Some of the additional policy actions modeled in this scenario include the proposed federal regulations for fossil fuel power plants, accelerated adoption of EVs in light-duty vehicle, bus, and freight truck markets, enhanced electric appliance standards, more stringent standards on oil and gas methane emissions, as well as extensions of the existing IRA tax credits beyond their legislated sunset dates.

Please see Supplementary Note 1 for more information on the current policy landscape and scenario design. A summary of modeled policies for each sector in both scenarios is included in Table 1, and detailed modeling assumptions underlying implementation of all policy representations in GCAM-USA-CGS are listed in Supplementary Notes 6–11.

We also assessed emissions projections from the two scenarios by varying assumptions on a few important drivers, including GDP, population growth, oil and gas prices, solar and wind costs, and the land sink carbon sequestration potential. Although these drivers do not capture the full range of potential drivers, they do provide a reasonable range of emissions projections that vary the technical and economic potential of our scenarios. Please see Supplementary Note 15 for a description of our assumptions in the sensitivity analysis.

### Supplementary information

Supplementary Information

## Data Availability

Comprehensive output data summaries are available at 10.5281/zenodo.10425458.

## Code availability

GCAM is an open-source community model available at http://github.com/JGCRI/gcam-core/releases (10.5281/zenodo.5093192). Input files used for this study are available at 10.5281/zenodo.10425458, and detailed model results can be obtained running this input file set using the release version of GCAM 6.0 available at the above web site. Results presented in this paper may differ in some minor aspects from those obtained using the release version of the model. Additionally, as noted in Supplementary Note 12, GHG emissions results were post processed to harmonize with the EPA inventory, and will differ from raw model results.
