# Supplementary material for: High-ambition climate action in all sectors can achieve a 65% greenhouse gas emissions reduction in the United States by 2035
Source: NPJ Clim Action. 2024 Jul 24;3(1):63. doi: 10.1038/s44168-024-00145-x (PMC11269174; doi:10.1038/s44168-024-00145-x)
Supplement: Supplementary file 1 — Supplementary Information [file 44168_2024_145_MOESM1_ESM.pdf]

Supplementary Information

High-ambition climate action in all sectors can achieve 65% greenhouse gas emissions reduction in the United States by 2035

Authors: Alicia Zhao[a,g], Kowan T.V. O'Keefe[a], Matthew Binsted[a,b], Haewon McJeon[c], Adriana Bryant[a], Claire Squire[a], Mengqi Zhang[d], Steven J. Smith[a,b], Ryna Cui[a], Yang Ou[e,f], Gokul Iyer[a,b], Shannon Kennedy[a], and Nate Hultman[a]

Affiliations:

[a] Center for Global Sustainability, University of Maryland, College Park, Maryland, USA

[b] Joint Global Change Research Institute, Pacific Northwest National Laboratory, College Park, Maryland, USA

[c] Graduate School of Green Growth & Sustainability, Korea Advanced Institute of Science and Technology, Daejeon, Korea

[d] Global Energy Monitor, Covina, California, USA

[e] College of Environmental Sciences and Engineering, Peking University, Beijing, China

[f] Institute of Carbon Neutrality, Peking University, Beijing, China

[g] Corresponding author, aszhao@umd.edu

Table of Contents

Supplementary Methods 3
Supplementary Note 1. Current policy landscape and scenario design 3
Supplementary Note 2. State tiering 5
Supplementary Note 3. Overview of GCAM-USA-CGS 7
Supplementary Note 4. Overview of modeling approach 8
Supplementary Note 5. Interactions between federal and state policies 9
Supplementary Note 6. Modeled IRA provisions 10
Supplementary Note 7. Core model assumptions 11
Supplementary Note 8. Electricity modeling assumptions 11
Supplementary Note 9. Transportation sector modeling assumptions 13
Supplementary Note 10. Building sector modeling assumptions 17
Supplementary Note 11. Industry and other sectors modeling assumptions 18
Supplementary Note 12. EPA inventory calibration 20
Supplementary Note 13. Global warming potential 20
Supplementary Note 14. Model validation 21
Supplementary Note 15. Sensitivity analysis 22
Supplementary Note 16. Bottom-up aggregation analysis 23
Supplementary Note 17. Land Use, Land-use Change, and Forestry 30
Supplementary Figures 31
Supplementary References 41

Supplementary Methods

Supplementary Note 1. Current policy landscape and scenario design

Recent federal action in the United States has strengthened emissions reduction potential. Together, the Inflation Reduction Act (IRA) and Bipartisan Infrastructure Law (BIL) provide over a trillion in funds toward clean energy tax credits, electrification and energy efficiency measures, methane emissions reductions, and the expansion of electric vehicle charging infrastructure. [6,7] Existing executive actions include the Corporate Average Fuel Economy (CAFE) standards, which mandate the fuel efficiency of passenger cars and light duty trucks, and New Source Performance Standards (NSPS) under the sections 111(b) and (d) of the Clean Air Act (CAA), which set pollution limits on fossil fuel electricity generation. Additionally, proposed rules would strengthen oil and gas methane regulations and phase down coal- and gas-fired electricity generation.[8,9]

Across sectors, subnational policies have the capability to build upon federal actions. States and cities are setting their own economy-wide emissions reductions goals and formulating policies to help them achieve their targets.[10,11] Within the power sector, state-level clean energy targets and local zoning ordinances can accelerate decarbonization of electricity, such as Maryland's renewable portfolio standard (RPS), which mandates that electricity suppliers must procure 50% of their electricity from renewable sources by 2030, and the city of Austin's target of carbon-free electricity by 2035.[12,13] Building sector mitigation can be hastened through state and city-level appliance standards and efficiency targets, such as legislation passed in the San Francisco Bay Area that bans new sales of nitric oxide (NO) and nitrogen dioxide (NO2)-emitting space heaters and water heaters, beginning with water heaters in 2027.[14] Transportation decarbonization measures include sales targets and incentives for phaseout of combustion engines, such as California's Advanced Clean Trucks (ACT) rule, which an increasing number of states are adopting, and investment in public transportation like the Indianapolis Public Transportation Corporation's (IndyGo) expansion of public bus routes and rollout of hybrid and electric buses.[15,16] Mandates for more stringent management of fugitive methane and hydrofluorocarbon (HFC) emissions alongside incentives for carbon capture utilization and storage (CCUS), like New Mexico's 2022 legislation limiting venting and flaring at oil and gas sites, can similarly use subnational levers for industrial emissions reductions.[3,17]

The *Current Policies* scenario includes existing federal policies – including several climate and energy provisions from the BIL and the IRA – and non-federal policies. Altogether, we find that these current policies can reduce emissions 45% below 2005 levels by 2035 (see

Supplementary Table 1 for a sectoral breakdown of emissions reductions). Existing state-level RPS, CAFE standards, and several tax credits from the IRA for renewable electricity generation, zero-emissions vehicles (ZEVs), and carbon capture and storage (CCS) are among the key policy drivers in this scenario. Detailed modeling assumptions for representation in GCAM-USA-CGS 6.0 for these and all other policies in the Current Policies scenario are shown in Supplementary Tables 2-6.

The IRA, along with additional policies and actions from Congress, the federal government, states, cities, and businesses, collectively provide a major boost to climate action in the United States. Yet these existing policies will not be enough on their own for the United States to meet its 2030 climate target and achieve ambitious reductions through 2035 and beyond. Thus, our *Enhanced Ambition* scenario models greenhouse gas (GHG) emissions reductions achievable under a comprehensive, "all-in" climate strategy that builds upon the policy framework in the *Current Policies* scenario with enhanced non-federal and federal action. Altogether, these actions have the potential to deliver a 52% reduction in GHG emissions from 2005 levels by 2030, thus fulfilling its 2030 NDC, and a 65% reduction by 2035. A sector-by-sector breakdown of the results for this scenario is shown in Supplementary Table 1 alongside results from our *Current Policies* scenario. Some of the additional policy actions modeled in this scenario include a full phaseout of unabated coal-fired electricity generation by 2030, accelerated adoption of ZEV in light-duty vehicle, bus, and freight truck markets, ramped up electric appliance standards, more stringent standards and oil and gas methane, as well as extensions of the existing IRA tax credits beyond their legislated sunset dates. The modeling assumptions underlying implementation of all policy representation in GCAM-USA-CGS in this scenario are listed in Supplementary Tables 2-6.

Supplementary Table 1. GHG emissions results by sector

**Sector/GHG**	**Emissions 2005 ($MMTCO_2e$)**	**Emissions 2020 ($MMTCO_2e$)**	**Emissions 2035 ($MMTCO_2e$)**		**Change from 2005 to 2035 ($MMTCO_2e$)**		**Change relative to 2005 (%)**		**Contribution to total reductions relative to 2005 (%)**	
			Current Policies	***Enhanced Ambition***	***Current Policies***	***Enhanced Ambition***	***Current Policies***	***Enhanced Ambition***	***Current Policies***	***Enhanced Ambition***
Electricity CO_2	2,417	1,457	629	86	-1,788	-2,331	-74%	-96%	60%	53%
Transport CO_2	1,869	1,581	1061	815	-808	-1,054	-43%	-56%	27%	24%

Industry CO_2	1,193	1,106	1149	974	-44	-219	-4%	-18%	1%	5%
Buildings CO_2	586	542	456	335	-130	-251	-22%	-43%	4%	6%
Other CO_2	67	32	28	21	-40	-47	-59%	69%	1%	1%
CH_4	852	807	766	568	-86	-284	-10%	-33%	3%	6%
N_2O	427	400	409	402	-18	-25	-4%	-6%	1%	1%
F-Gases	138	180	111	92	-28	-46	-20%	-33%	1%	1%
Direct Air Capture	0	0	0	-31	0	-31	0	N/A	0%	1%
Land sink	-854	-853	-882	-926	-28	-73	3%	8%	1%	2%
**Net GHG Total**	**6,697**	**5,252**	**3,728**	**2,339**	**2,969**	**4,357**	**44%**	**65%**	**100%**	**100%**

Supplementary Note 2. State tiering

State-level climate action in the United States varies considerably from across the country. For example, some states aim to achieve 100% ZEV sales in light duty vehicle (LDV) markets by 2035, while other states have less ambitious ZEV sales targets, and some lack ZEV sales targets entirely. Therefore, to account for the unequal ambition and urgency with which states implement policies and actions to reduce emissions, and to facilitate our scenario analysis, we group states into three different tiers in the *Enhanced Ambition* scenario.

State-level tiering reflects the propensity of a state to take further climate action based on the strength of their past and current climate policies. Tier 1 states have been leading the way on climate action in the United States and we assume that they will adopt a full range of climate policies in ambitious decarbonization scenarios. Tier 2 states have some policies in place, but tend to move slower than Tier 1 states on climate action. Thus, we assume that they will adopt some of the additional climate policies in ambitious decarbonization scenarios, although at a slower rate than Tier 1 states. The rest of the states, categorized as Tier 3, have taken limited steps to advance climate action, and are assumed to continue at a slow pace. We therefore assume limited additional policy action in ambitious

decarbonization scenarios, and typically on slower time scales than we assume for Tier 1 and Tier 2 states.

Some of the markers that we use to categorize states as Tier 1, Tier 2, or Tier 3 include their on-the-books climate policies (including RPS, EERS), vocal leadership in support of climate action, the ambition of their emission reduction targets or standards, and their memberships in leadership organizations like the U.S. Climate Alliance.

- **Tier 1 states:** California, Colorado, Connecticut, Delaware, the District of Columbia, Hawaii, Illinois, Maine, Maryland, Massachusetts, Minnesota, New Hampshire, New Jersey, New Mexico, New York, Oregon, Pennsylvania, Rhode Island, Vermont, and Washington
- **Tier 2 states**: Arizona, Iowa, Michigan, Missouri, Nevada, North Carolina, Ohio, Virginia, and Wisconsin
- **Tier 3 states:** Alabama, Alaska, Arkansas, Florida, Georgia, Idaho, Indiana, Kansas, Kentucky, Louisiana, Mississippi, Montana, Nebraska, North Dakota, Oklahoma, South Carolina, South Dakota, Tennessee, Texas, Utah, West Virginia, and Wyoming

The tier system is implemented as differences in the speed of policy adoption and as differences in the target level. For example, tiering for state-level EV sales targets is based on the speed of uptake. Tier 1 states meet the Advanced Clean Cars II and Advanced Clean Trucks targets, while Tier 2 and Tier 3 states achieve the same targets but lag by 5 and 10 years, respectively. For RPS, where implementation is based on differences in the target level, Tier 1 states achieve 75% renewables, Tier 2 states achieve 55% renewables, and Tier 3 states achieve 20% renewables. Targets for the different tiers are estimated based on high-achieving states in each tier. For details on state-level policy assumptions please see Supplementary Tables 3-6.

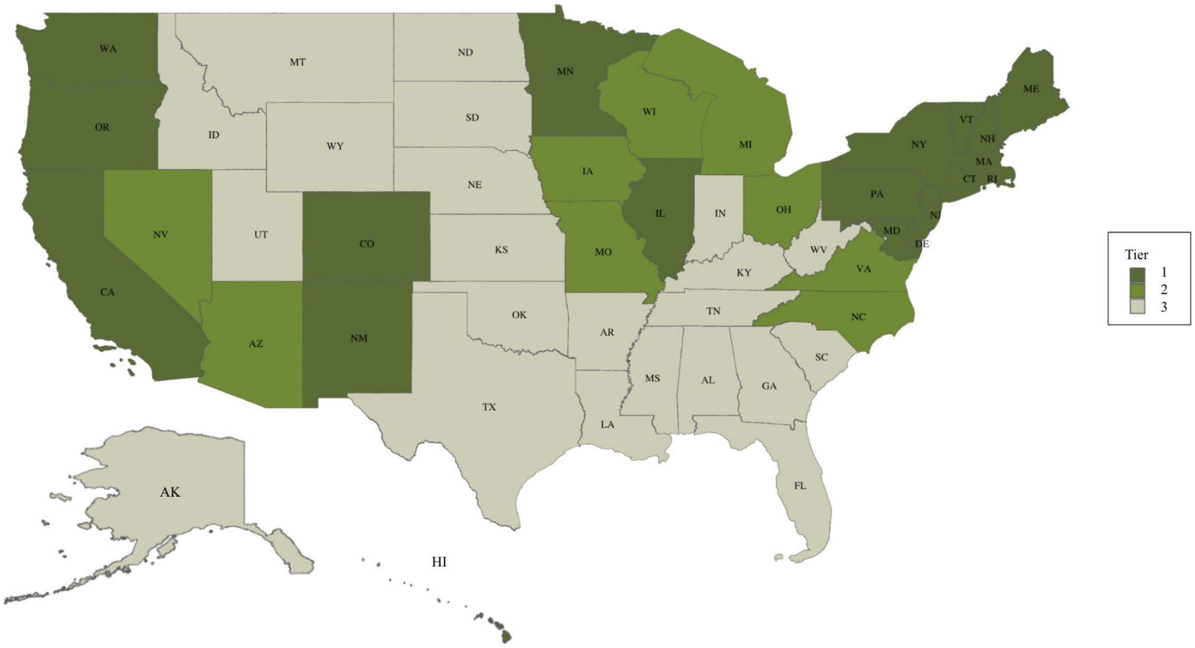

Supplementary Figure 1. State tiers used in the *Enhanced Ambition* scenario.

Supplementary Note 3. Overview of GCAM-USA-CGS

Our analysis uses a version of the open-source Global Change Analysis Model (GCAM) to estimate the aggregate impact of federal and non-federal climate policies and actions on economy-wide emissions reductions in the United States. Specifically, we use GCAM-USA, a state-level version of GCAM. We refer to the version of GCAM-USA used in this study as GCAM-USA-CGS.

GCAM is an integrated assessment model (IAM) that covers the energy, land, water, climate, and socioeconomic systems. The global version of GCAM groups the world's countries into 32 geopolitical regions with representation of the energy and socioeconomic systems for each region. The United States is one of the 32 regions. GCAM represents the global climate system, and uses 235 water basins and 384 land regions to represent global water and land systems. GCAM tracks emissions and sinks of carbon dioxide (CO_2), 16 other GHGs, and several air pollutants.

The state-level version of GCAM used in this analysis, GCAM-USA, disaggregates the U.S. energy and economy components into 50 states and the District of Columbia while maintaining the same level of detail as GCAM for water and land sectors. The energy system in GCAM-USA has representations of depletable primary sources such as coal, gas,

oil, and uranium, in addition to renewable resources such as biomass, hydropower, solar, wind, and geothermal. Energy transformation processes like oil refining and electricity generation are represented at the state-level in GCAM-USA. These energy carriers, in turn, are used to deliver services to state-level end users in the buildings, transportation, and industrial sectors. The electric power sector includes representations of a range of power generation technologies, including those fueled by fossil fuels and bioenergy (with and without CCS), renewables, and nuclear power.

GCAM-USA is a market equilibrium model. The model solves for equilibrium in each period by finding a set of market prices such that supplies and demands are equal to one another in all markets as the actors in the model adjust the quantities of the commodities they buy and sell. GCAM operates in 5-year time-increments, with each new period starting from the conditions that emerged in the last, with most technologies vintaged such that a portion of existing stocks at any point carry over into future time periods.

GCAM-USA-CGS is based on the open-source release of GCAM-USA 6.0.[18] GCAM-USA-CGS has been updated for the purposes of this study to reflect changes such as the most recent estimates of future renewable energy costs.[19] The model is also calibrated to the latest non-CO_2 marginal abatement cost curves from the U.S. Environmental Protection Agency (EPA).[20]

Supplementary Note 4. Overview of modeling approach

Policy representation in our modeled scenarios builds upon bottom-up aggregation tools and data analysis to evaluate and quantify the impacts of policies and climate actions in isolation and within specific sectors. Throughout, we took care to avoid potential double counting of potential emissions reduction drivers from nested governance levels. We then used this information in GCAM-USA-CGS to estimate the economy-wide implications of associated policies. We use a modeling approach consistent with previous analyses, including Accelerating America's Pledge (2019), An All-In Climate Strategy Can Cut U.S. Emissions by 50% by 2030 (2021), Blueprint 2030 (2021), and An All-In Pathway to 2030 (2023).[1,2,4,21,22]

All modeled policies in GCAM-USA-CGS are implemented at the state and/or national levels. Policies and actions from city governments, businesses, and institutions were aggregated to the state-level or assumed to be embedded within or supportive of the state and/or national level policy representation in the model, and therefore not explicitly modeled to remove risk of double counting potential emissions reductions. Detailed descriptions of

policy representation in GCAM-USA-CGS can be found in Supplementary Tables 3-6. and documentation of the bottom-up aggregation processes across different sectors can be found in Supplementary Note 16.

Model parameters in GCAM-USA-CGS varied according to information from our bottom-up aggregation analysis or changed directly for policy drivers where bottom-up aggregation was either not feasible or not necessary in the case of small-scale potential impacts. The purpose of this analysis is to assess the national emissions reduction potential in the United States for the policies modeled in our scenarios. Accordingly, non-federal policies and actions are only modeled to the extent that doing so would have a meaningful impact on the national-level emissions outcome. In some policy areas, we did not specify state-level variation in policy implementation – for example, with bus electrification – as assessment of the national-level emissions impact does not require state-level precision.

The policies and actions used in our analysis were compiled up until August 2023. New or changed policies and actions taken after this point are not included in this analysis.

Supplementary Note 5. Interactions between federal and state policies

In our study, state-level policies were largely modeled to enhance federal policies. Generally, we modeled federal actions and incentives first, and then layered on other actions. We took care to avoid double counting in cases where it may be applicable. For example, in modeling LDV electrification, we first implement the state- and federal-level EV tax credits to see how much electrification would be driven by changes in cost. Then, we layer on the state-level sales targets to close the gap for the states that are still not meeting their targets. Some states already meet their targets in certain years with the tax credits, so we do not implement any additional sales in this case. After implementing these targets, our EV sales shares come close to the sales required by the proposed tailpipe emissions standards; thus, we do not explicitly model the standards, but assume that they would support states in achieving their targets. In the power sector, we first model the IRA clean energy tax credits and investments, and any federal power plant regulations. We then layer on state-level RPS to deploy any additional renewable generation that is needed. For the PJM grid region, we double check the power sector emissions to see if they comply with RGGI targets. If they already exceed the RGGI targets, then we do not model any additional policies. If they fall short of the RGGI targets, then we model an emissions constraint for the power sector on top of the other bottom-up policies.

Additionally, in cases where there is ambiguity around the interpretation of certain policies, we opted for the climate-smart interpretation of these provisions. In interpreting BIL, for example, some of the provisions could potentially fund highway repairs instead of EV infrastructure; however, we chose to assume that the funding would go toward EV infrastructure.

We also note that the modeling assumes full implementation of binding targets and incentives. In reality, the presence of a binding target does not necessarily entail that the target will be met. Furthermore, in the case of the IRA provisions, full implementation of the investments may not be achieved if non-federal actors do not take advantage of these provisions or use them appropriately.

Supplementary Note 6. Modeled IRA provisions

Both scenarios include over 20 IRA provisions, which are listed below. Detailed assumptions for these policies are described in Supplementary Tables 3-6.

- Section 13101: Production tax credit (PTC)
- Section 13102: Investment tax credit (ITC) extension
- Sections 13701 & 13702: New clean electricity PTC and ITC
- Section 13302: Residential clean energy credit
- Section 13015: PTC for existing nuclear
- Section 50144: Energy infrastructure reinvestment financing
- Section 13104 – 45Q: Extension of credits for captured CO_2
- Section 13401 – 30D: Clean vehicle credit
- Section 13404: Alternative refueling property credit
- Section 13403 – 45W: Commercial clean vehicle credit
- Sections 13201, 13202, and 13203: Extension of incentives for biofuels
- Section 13303: Energy efficient commercial building deduction
- Sections 13301 – 25C, 13304, and 50121: Energy efficient home improvement credit, Energy efficient home credit, and Home energy efficiency credit
- Section 51022: High efficiency home rebate program
- Section 13204 – 45V: Production credit for clean hydrogen
- Section 13501 – 48C: Manufacturing investment tax credit for advanced energy projects
- Section 50161: Advanced industrial facilities deployment program
- Section 60113: Methane emissions reduction program

Supplementary Note 7. Core model assumptions

The results of this study depend on many assumptions about how the U.S. and the world might evolve in the future. This study uses a set of core assumptions for drivers including economic growth, population growth, fossil fuel prices, demand impacts of the COVID-19 pandemic, and technology costs (Supplementary Table 2). Our core assumptions draw from a set of data sources that are referenced in other parts of this appendix, for example U.S. Energy Information Agency (EIA)'s Annual Energy Outlook (AEO)[23] and Rhodium Group.[24] Economic impacts associated with COVID-19 in 2020 and subsequent recovery in the following years have also been incorporated into these assumptions.

Supplementary Table 2. Core assumptions for GCAM-USA-CGS analysis

**Drivers**	**Scenario assumptions**
Economic Growth	Overall gross domestic product (GDP) decreases by 3.5% year-on-year on average in 2020, then increases by 2.1% per year on average through 2035.
Population Growth	Population grows by 0.61% per year on average through 2035.
Fuel Prices	Gas price is assumed to drop by 19.5% year-on-year in 2020, increase by 89% in 2021, then decrease at an average rate of 7.1% per year through 2025. Prices increase 0.1% on average between 2025 and 2035.<br><br>Oil price is assumed to drop by 33.9% year-on-year in 2020, increase by 78.4% in 2021. Prices increase at an average rate of 0.7% per year between 2021 and 2035.
Transportation Energy Demand	Transport sector energy demand is assumed to decrease by 6.9% from 2015 levels in 2020, with recovery through 2030.
Industry Energy Demand	Industry sector energy demand is assumed to decrease by 3.1% from 2015 levels in 2020, with recovery through 2030.
Buildings Energy Demand	Buildings sector energy demand is assumed to decrease by 1.9% from 2015 levels in 2020, with recovery through 2030.
Technology Costs	Technology costs are updated with the National Renewable Energy Laboratory (NREL) Annual Technology Baseline 2022 assumptions.[19]

Supplementary Note 8. Electricity modeling assumptions

In this and subsequent sections the modeling assumptions are provided in tables that identify the type of policy (federal vs. non-federal), the specific portion of the policy (where relevant), and column with a description of the assumptions used in the *Current Policies* scenario, followed by a description (where different) of how the policy is modeled in the *Enhanced Ambition* scenario.

Supplementary Table 3. Implementation of policy assumptions for the electricity sector in GCAM-USA-CGS

Type of Policy	Modeled Policy	*Current Policies* Scenario	*Enhanced Ambition* Scenario
Federal – IRA	Section 13101: PTC	Modeled as a $26/MWh subsidy for solar, wind and geothermal technologies through 2024.[25] We assume that all projects pay prevailing wages. A 7.5% reduction in the credit value is assumed due to the transferability provision.	
	Section 13102: ITC extension	Modeled as a 30% subsidy for offshore wind and storage technologies through 2024,[25] with the simplifying assumption that all projects pay prevailing wages. A 7.5% reduction in the credit value is assumed due to the transferability provision.	
	Sections 13701 and 13702: New clean electricity PTC and ITC	Modeled in the same way as sections 13101 and 13102 through 2030, with phasedown after 2030.	In addition to the assumptions under *Current Policies*, the subsidies are extended through 2035.
	Section 13302: Residential clean energy credit	Modeled by updating the rooftop ITC, which results in an additional 0.7GW/yr increase in electricity generation from rooftop solar photovoltaic (PV) on the lifetime of the credit through 2035.[25]	
	Section 13015: PTC for existing nuclear	Modeled as a $15/MWh subsidy for nuclear technologies through 2030, with the simplifying assumption that all projects pay prevailing wages. [25]<br>We assume that these incentives, in combination with non-federal incentives and ZEV credits, prevent the economic retirement of nuclear plants. As such, we model Georgia Vogtle units 3&4 coming online by 2025, and maintain nuclear capacity at today's levels.	
	Section 50144: Energy infrastructure reinvestment financing	Modeled as $250 billion in loans and guarantees used to accelerate the retirement of coal-fired power generation and fund the construction of renewable electricity-generating capacity.[25] We estimate this to accelerate the retirement of 38 GW of additional coal-fired capacity beyond already-scheduled retirements by 2030.	
	Section 13104 - 45Q: Extension of credits for captured CO_2	Credits for CO_2 captured by projects that commence construction between 2023 and 2032 are applied to CCS technologies at a value of $51/ton. [25] These credits assume that half of projects meet prevailing wage and apprenticeship requirements, but do not assume that all projects meet these requirements and receive the full credit value available ($85/ton). CCS technology deployment endogenously responds to these subsidies.	Credits for CO_2 captured by projects that commence construction between 2023 and 2032 are applied to CCS technologies at a value of $68/ton. [25]These credits assume that half of projects meet prevailing wage and apprenticeship requirements, but do not assume that all projects meet these requirements and receive the full credit value available ($85/ton). CCS technology deployment endogenously responds to these subsidies.
Federal – Regulations	CAA section 111(b) Standards for New Stationary Combustion Turbines	Not explicitly modeled in this scenario.	Proposed federal standards (May 2023)under CAA section 111(b) require at least 90% CCS for any new baseload natural gas builds in all states by 2035.[26] This was modeled by reducing new baseload natural gas power plants without CCS starting in 2025 and eliminating new baseload natural gas power plants without CCS by 2030. This reflects an expectation that investors

			will change their behavior in anticipation of the 2035 policy target, especially in light of the 111(d) emissions guidelines described below. Retention and investment in low capacity factor peaking plants were assumed to be supportive of these measures and were not explicitly modeled.
	CAA section 111(d) Emission Guidelines for Existing Fossil Fuel-Fired Sources	Not explicitly modeled in this scenario.	Proposed federal standards (May 2023) under CAA section 111(d) for large, frequently used combustion turbines include two pathways: 96% low-GHG hydrogen in 2038 or 90% CCS in 2035.[26] This was modeled by mandating that all high-capacity factor natural gas power plants either retrofit with CCS by 2035 or cease operating (as determined by the economics of retrofitting). EPA is taking comment on standards for lower capacity factor other stationary combustion turbines, so low capacity factor peaking plants were not subject to the retrofit requirements.
Non-federal	RPS	Current state-level RPS targets are modeled. City- and utility-level goals were assumed to be supportive of these state-level targets and additional only in cases where a higher percentage is targeted. These were implemented by setting a minimum % of total electricity load to be met by renewable generation.	RPS targets of at least 75% by 2035 for Tier 1 states, 55% for Tier 2 states, and 20% for Tier 3 states are assumed. City- and utility-level goals were assumed to be supportive of these state-level targets and additional only in cases where a higher percentage is targeted. These were implemented by setting a minimum % of total electricity load to be met by renewable generation.
	Cap and Trade	The Regional Greenhouse Gas Initiative (RGGI) is modeled as a 30% reduction in power sector emissions below 2020 levels by 2030 in participating states.[27]	
Coal phaseout		Not explicitly modeled in this scenario	Coal is phased out by 2030 due to a combination of market forces, state coal-exit policies, and regulatory compliance costs. This was modeled by setting a national constraint on coal power to reach zero by 2030, and by prohibiting the buildout of new coal plants in all states.

Supplementary Note 9. Transportation sector modeling assumptions

Supplementary Table 4. Implementation of policy assumptions for the transportation sector in GCAM-USA-CGS

Type of Policy	Modeled Policy	*Current Policies* Scenario	*Enhanced Ambition* Scenario

Federal – IRA	Section 13401 - 30D: Clean vehicle credit	This tax credit has a maximum value of $7,500 with an EV being eligible for half of the credit if its battery meets domestic assembly requirements and other half of the credit is contingent upon a specific share of the minerals used in the battery being sourced for North American or other free trade countries.[25] We assume that the U.S. auto manufacturing sector will reorient itself so that all new EVs produced by 2030 will meet these requirements, and that by 2025, half of EVs sold will meet these requirements. If the car meets the battery assembly and mineral sourcing requirements, a consumer can receive the full value of the tax credit provided that their income does not exceed the income eligibility threshold and that the sales price of the car does not exceed manufacturer's suggested retail price (MSRP) eligibility thresholds. We find that 89% of Americans meet the income requirement and further assume that they would only purchase EVs that meet the MSRP threshold. Altogether, this yields an EV tax credit with an effective value of $6,673, implemented as a capital cost reduction. We assume that for the 2031-2035 model period that the tax credit takes on a value 40% of the 2030 value because it is scheduled to expire in 2032.	In addition to the assumptions under *Current Policies*, the tax credit is extended to be available through 2035.
	Section 13404: Alternative refueling property credit	This credit is assumed to be a $1,000 property credit available for LDV charging infrastructure for individuals in rural and low-income census tracts. [25] Based on census data, 17.4% of Americans live in counties that are either rural or low-income, so the $1,000 property credit is modeled as a weighted average national subsidy of $174 for capital infrastructure cost for EVs. We assume that for the 2031-2035 model period that the tax credit takes on a value 40% of the 2030 value because it is scheduled to expire in 2032.	In addition to the assumptions under *Current Policies*, the tax credit is extended to be available through 2035.
	Section 13403 - 45W: Commercial clean vehicle credit	This tax credit is modeled as a $40,000 capital cost reduction for electric heavy duty freight trucks, and a $7,500 capital cost reduction for electric medium duty and light duty freight trucks.[25] We assume that for the 2031-2035 model	In addition to the assumptions under *Current Policies*, the tax credit is extended to be available through 2035.

		period that the tax credit takes on a value 40% of the 2030 value because it is scheduled to expire in 2032.	
	Sections 13201, 13202, and 13203: Extension of incentives for biofuels	Implemented as subsidies in 2025 for biodiesel, cellulosic ethanol, Fischer Tropsch (FT) biofuels, cellulosic ethanol with CCS, and FT biofuels with CCS.[25] We assume that jet fuel is the first market for FT biofuel, and FT biofuels therefore receive the aviation fuel credit.	In addition to the assumptions under *Current Policies*, the tax credit is extended to be available through 2035.
Federal – BIL	Section 11401 and 11403: Grants from charging and fueling infrastructure, Carbon reduction program, and National Electric Vehicle Formula Program	BIL's $10.7 billion investment in LDV EV charging infrastructure is implemented as an $802 reduction in per vehicle charging infrastructure cost, based on modeled vehicle fleet size in GCAM-USA-CGS, for model periods 2025 and 2030.[7]	
	Section 11115 and 11403: Congestion mitigation and air quality improvement program, and Carbon reduction program	BIL's $4.24 billion investment in medium- and heavy-duty truck EV charging infrastructure is implemented as a $9,211 reduction in per vehicle charging infrastructure cost, based on fleet size in GCAM-USA-CGS, for model periods 2025 and 2030.[7]	
	Sections 71101 and 30018: Clean school bus program and Grants for buses and bus facilities	BIL's $5 billion investment in school bus electrification is implemented as a $25,000 reduction in per vehicle purchase cost for model periods 2025 and 2030. A $2.625 billion investment in transit bus electrification is implemented as a $29,167 reduction in per vehicle purchase cost for model periods 2025 and 2030.[7]	
Federal – Regulations	CAFE standards for LDVs	Internal combustion engine GHG performance standards are modeled to reflect efficiency improvement rates from recently updated CAFE so that nationally, fuel efficiency reaches 166 gCO_2/mi for new passenger cars and 219 gCO_2/mi for new SUVs by 2030.[28] Note: these are based on the National Highway Traffic Safety Administration (NHTSA) minimum standard and are not inclusive of ZEVs.	Federal internal combustion engine GHG performance standards are improved so that nationally, fuel efficiency reaches 143 gCO_2/mi for new passenger cars and 193 gCO_2/mi for new SUVs by 2030.
	GHG emissions standards for freight trucks	Internal combustion engine GHG performance standards are modeled to reflect efficiency improvement rates existing GHG emissions standards for	Same as in the *Current Policies* scenario through 2025. Efficiency is assumed to improve further by 5% from 2026-2030 and again from 2031-2035.

		heavy duty gasoline- and diesel-powered engines.[29]	
	Accelerated retirement of old, inefficient LDVs	Not explicitly modeled in this scenario.	Modeled as a “cash-for-clunkers” style program that accelerates the retirement of LDVs produced in 2015 and prior, effectively reducing the share of the 2015 and prior vintage cars remaining on the road in 2030 from 28% to 18%.
Non-federal	LDV ZEV sales mandates and targets	California and the 10 other states that have adopted ZEV sales targets consistent with California's Advanced Clean Cars (ACC) II legislation are assumed to achieve their passenger car sales target of 68% electric in 2030 and 100% in 2035. [30] Additionally, the 5 states that have only adopted legislation consistent with California’s ACC I legislation are modeled to have ZEV sales reach 22% in 2025.	Tier 1 states are assumed to achieve ZEV sales shares equivalent to targets set by California. Tier 2 and Tier 3 states are assumed to achieve these sales shares but on a delayed schedule, 3 years later (Tier 2 states) and 6 years later (Tier 3 states) than Tier 1 states.
	LDV ZEV incentives	Major existing incentives for LDV ZEVs at the state-, utility-, and district-level from the Alternative Fuels Data Center are modeled at the state-level as reductions in per vehicle capital cost. Altogether, these are equivalent to a national average capital cost reduction for LDV EVs of $826 per vehicle.	
	Freight truck ZEV sales mandates and targets	California and 12 other states are assumed to achieve sales targets for electric trucks through 2035 consistent with California’s ACT legislation.[31]	Tier 1 states are assumed to achieve ZEV sales shares equivalent to targets set by California. Tier 2 and Tier 3 states are also assumed to achieve these sales shares but on a delayed schedule, 3 years later (Tier 2 states) and 6 years later (Tier 3 states) than Tier 1 states.
	Bus ZEV incentives and sales targets	Not explicitly modeled in this scenario.	A combination of federal and non-federal investments and fleet procurement targets lead to 100% electrification of new bus sales in 2030. This was modeled by raising the national-level sales shares to reach 100% electric by 2030.
	Vehicle miles traveled (VMT) reductions	Not explicitly modeled in this scenario.	Federal investment, state and local planning lead to annual average per capita passenger transportation demand reductions ranging from 0.75% to 1.25% in all states from 2025-2035 (consistent with current ambition in Tier 1 states).[32,33] Annual average per capita VMT reductions were modeled as state-level service demand reduction rates for passenger mode transit.

Supplementary Note 10. Building sector modeling assumptions

Supplementary Table 5. Implementation of policy assumptions for the buildings sector in GCAM-USA-CGS

<table>
<tr><th>Type of Policy</th><th>Modeled Policy</th><th>Current Policies Scenario</th><th>Enhanced Ambition Scenario</th></tr>
<tr><td rowspan="3">Federal – IRA</td><td>Section 13303: Energy efficient commercial building deduction</td><td>This provision is estimated to reduce commercial HVAC costs by 3%.[25] This was modeled as a 3% subsidy for commercial high-efficiency heating and cooling technologies in 2025 and 2030.</td><td>In addition to the assumptions under Current Policies, the subsidies are extended through 2035.</td></tr>
<tr><td>Sections 13301 - 25C and 13304 and 50121: Energy efficient home improvement credit, Energy efficient home credit, and Home energy efficiency credit</td><td colspan="2">Modeled by improving shell efficiency in residential buildings based on the AEO 2022 “Alternative Policies – Extended Credit” case.[25,34]</td></tr>
<tr><td>Section 51022: High efficiency home rebate program</td><td>Modeled as a subsidy to high-efficiency technologies in residential buildings in 2025 and 2030. Two-thirds of consumers are assumed to be eligible for this credit, so this was implemented as a weighted average across all consumers with the effective value of the credit modeled to be 66% of each of the following: $1,750 to electric heat pump water heaters, $4,000 to electric heat pumps for space heating, $420 to electric ovens, $420 to electric heat pump clothes dryers, $1,600 for high-efficiency air conditioning.[25]</td><td>In addition to the assumptions under Current Policies, the subsidies are extended through 2035.</td></tr>
<tr><td rowspan="2">Non-federal</td><td>Energy efficiency standards (EERS)</td><td>Current state-level EERS were modeled by reducing state-level building service demands. However, the energy savings yielded are insignificant at the national level.</td><td>Heightened EERS and building codes were modeled by reducing state-level building service demands. This leads to national energy savings of up to 7% for residential buildings and up to 10.7% for commercial buildings by 2035. See Section 5 for more details.</td></tr>
<tr><td>Electrification incentives</td><td>Not explicitly modeled in this scenario.</td><td>Zero emissions appliance standards were modeled by driving space heating and water heating sales to 100% electric by 2030 in Tier 1 states and 2035 in Tier 2 states.[14] The residential and commercial buildings sectors each achieve 67% electrification by 2035. See Section 5 for more details.</td></tr>
</table>

Supplementary Note 11. Industry and other sectors modeling assumptions

Supplementary Table 6. Implementation of policy assumptions for industry and other sectors in GCAM-USA-CGS

Type of Policy	Modeled Policy	*Current Policies* Scenario	*Enhanced Ambition* Scenario
Federal - IRA	Section 13104 - 45Q: Extension of credits for captured CO_2	Credits for CO_2 captured by projects that commence construction between 2023 and 2032 are applied to relevant technologies at a value of \$51/ton for CCS. [25] These credits assume that half of projects meet prevailing wage and apprenticeship requirements, but do not assume that all projects meet these requirements and receive the full credit value available (\$85/ton). Existing credits for captured CO_2 at \$85/ton is implemented as a subsidy for cement and ethanol CCS respond to these subsidies endogenously, resulting in 9 $MtCO_2$ annual sequestration by 2035.	As a result of this subsidy, it is assumed that all states with cement production install CCS capability for 40% of cement produced by 2035, consistent with California's 40% goal.[35] This subsidy is also assumed to result in sequestration levels from ethanol CCS consistent with Rhodium Group's analysis by 2035.[36] A small amount of CCS in the paper and pulp industry is modeled, well below the U.S. share of global potential identified in the literature.[37] Sequestration was exogenously specified across various industrial sectors, resulting in 85 $MtCO_2$ annual sequestration.
	Section 13204 - 45V: Production credit for clean hydrogen	Modeled as different subsidies to hydrogen technologies depending on their carbon intensities.[25] Fossil hydrogen without CCS is assumed to claim 45Q instead. 50% of projects are assumed to pay prevailing wages.	
	Section 13501 - 48C: Manufacturing investment tax credit for advanced energy projects	Designates \$10 billion for industrial and manufacturing facilities aiming to equip themselves with technology to curtail GHG emissions.[25] This was modeled by specifying electrification rates aligned with an Energy Innovation analysis on low-temperature heating in the industrial sector.[38]	
	Section 50161 - Advanced industrial facilities deployment program	Designates \$5.8 billion towards advanced industrial technology at energy intensive industrial and manufacturing facilities.[25] This was assumed to result in faster equipment stock turnover for fossil fuels, modeled by shortening the average lifetime of existing industrial facilities.	In addition to the assumptions under *Current Policies*, we assume that new coal is not used as a fuel source in all industries.
	Section 60113: Methane emissions reduction program	This provision has a fee of \$1,500/tCH_4 (\$60/tCO_2e) on fugitive methane, modeled to reduce 2.92 $MtCH_4$ (73 $MTCO_2e$) in the oil and gas sector, using the EPA's MAC curves for methane.[20,25] Because this fee only applies to sources	An economy-wide methane fee of \$1,500/tCH_4 (\$60/tCO_2e) was modeled, using the EPA's MAC curves for methane and the activity reduction drivers for oil & gas, coal, and waste as calculated in our previous report on methane

		covered under the EPA's GHG Reporting Program, only 39% of the emissions reductions are assumed to be achieved [39], resulting in a reduction of 2.04 $MtCH_4$ (51 $MtCO_2e$) below 2020 levels by 2030. This reduction is also inclusive of the recently finalized oil and gas methane regulations that we assume delivers all reductions achievable from the EPA's MAC curves at a cost below $0/$tCO_2e$ for this sector.	emissions reduction potential in the U.S.[40] This resulted in methane reductions of 8.72 $MtCH_4$ (212 $MtCO_2e$) below 2020 levels by 2035.
HFCs		National HFC phasedown is implemented consistent with the American Innovation and Manufacturing (AIM) Act, reducing emissions up to 47% below 2020 levels by 2035 (consistent with analysis and modeling results developed by California Air and Resources Board (CARB)).[1]	National HFC phasedown is implemented consistent with the AIM Act. Tier 1 states achieves additional reductions through more comprehensive measures including Significant New Alternatives Policy (SNAP) and Refrigerant Management Programs (RMP) programs, reducing emissions up to 54% below 2020 levels by 2035 (consistent with analysis and modeling results developed by CARB).[12]
Land use, land-use change and forestry (LULUCF)		LULUCF emissions for CO_2 and N_2O are specified exogenously and adapted from *America Is All In*'s lands sector report.[41] *Current Policies* assumes full implementation of roughly $42 billion of current state and federal policies in agriculture and forestry practices, including BIL and IRA. This results in a carbon sink of 765 $MtCO_2e$ from forestry and 31 $MtCO_2e$ from agriculture, which increases sequestration potential by 30 $MtCO_2e$ from 2020 levels. For CH_4 emissions assumptions, please see *Section 60113: Methane emissions reduction program* above.	LULUCF emissions for CO_2 and N_2O are specified exogenously and adapted from *America Is All In*'s lands sector report.[41] *Enhanced Ambition* assumes $160 billion in investments in climate-smart policies resulting from enhanced state-level action. This results in a carbon sink of 778 $MtCO_2e$ from forestry and a carbon sink of 62 $MtCO_2e$ from agriculture, which increases sequestration potential by 74 $MtCO_2e$ from 2020 levels. For CH_4 emissions assumptions, please see *Section 60113: Methane emissions reduction program* above.
Economy-wide GHG targets		Not explicitly modeled in this scenario.	Tier 1 states are assumed to achieve their near- and long-term economy-wide GHG targets. Economy-wide GHG constraints were modeled for states that were more than 1 $MtCO_2e$ away from their target in 2035, including California, Washington, and New Jersey.
Direct air carbon capture and		Not explicitly modeled in this scenario.	DACCS is included as an additional mitigation option, resulting in 31 $MtCO_2$

[1] Emissions impacts from national and state-level HFC regulations were derived from a short-lived climate pollutant tool developed by CARB and extrapolated to additional states. We used the tool's Kigali phasedown scenario as a proxy for the impact of the AIM Act.

storage (DACCS)		of annual removals by 2035. This level of removal is consistent with announced DACCS facilities in the United States.[42]

Supplementary Note 12. EPA inventory calibration

For both scenarios, we calibrated emission results through the year 2020 to historical GHG emissions from the 1990-2021 EPA inventory report.[43] 2020 is a model year in GCAM-USA-CGS, which operates in 5-year time steps. However, we note that net GHG emissions from the 1990-2021 EPA inventory report increased by 6.4% between 2020 and 2021, so the reductions needed relative to 2021 will be somewhat higher than reductions relative to 2020, which is the base-year used in the paper.

Supplementary Note 13. Global warming potential

We use the 100-year global warming potential (GWP) from the IPCC Fifth Assessment Report (AR5) to convert non-CO_2 GHGs into CO_2 equivalents, as this is aligned with the latest EPA inventory, and will be required by the end of 2024 for UNFCCC reporting to reflect updated science .[44] However, GWP values from the Fourth Assessment Report (AR4) are commonly used to make these conversions. While there is slight variation in total emissions reductions if we use AR4 instead of AR5, these variations do not make a significant difference in our conclusions. Supplementary Table 7 below compares the reductions achieved under different GWPs under AR5 and AR4.

Supplementary Table 7. Economy-wide GHG emissions under *Current Policies* and *Enhanced Ambition* using AR4 and AR5 GWP values

Scenario	GWP	2005 Emissions ($MtCO_2e$)	2035 Emissions ($MtCO_2e$)	Emissions reduction from 2005 levels	Emissions reduction from 2005 levels (%)
Current Policies	AR5	6,697	3,728	2,969	44.3%
Current Policies	AR4	6,666	3,691	2,975	44.6%
Enhanced Ambition	AR5	6,697	2,339	4,357	65.1%
Enhanced Ambition	AR4	6,666	2,327	4,339	65.1%

Supplementary Note 14. Model validation

To validate the emissions estimates used in our *Current Measures* scenario, we compared our results with values found in previous studies that include the impact of the IRA on future energy emissions in the electricity, buildings, industry, and transportation sectors (Fig. S2). We used emissions estimates from five economy-wide models (EPS-EI, MARKAL-NETL, NEMS-RHG, REGEN-EPRI, and RIO-REPEAT) used in Bistline et al.'s 2023 multi-model comparison paper and the reference case projection in the EIA's 2023 Annual Energy Outlook.[34,45] In 2030, other projections estimate that emissions in these sectors will range from 3,311 - 4,011 Mt-CO_2e/yr, similar to our *Current Measures* total of 3,598 Mt-CO_2e/yr. Similarly, our 2035 emissions estimate of 3,314 Mt-CO_2e/yr falls between the other model projections, which range from 2,653 - 3,903 Mt-CO_2e/yr.

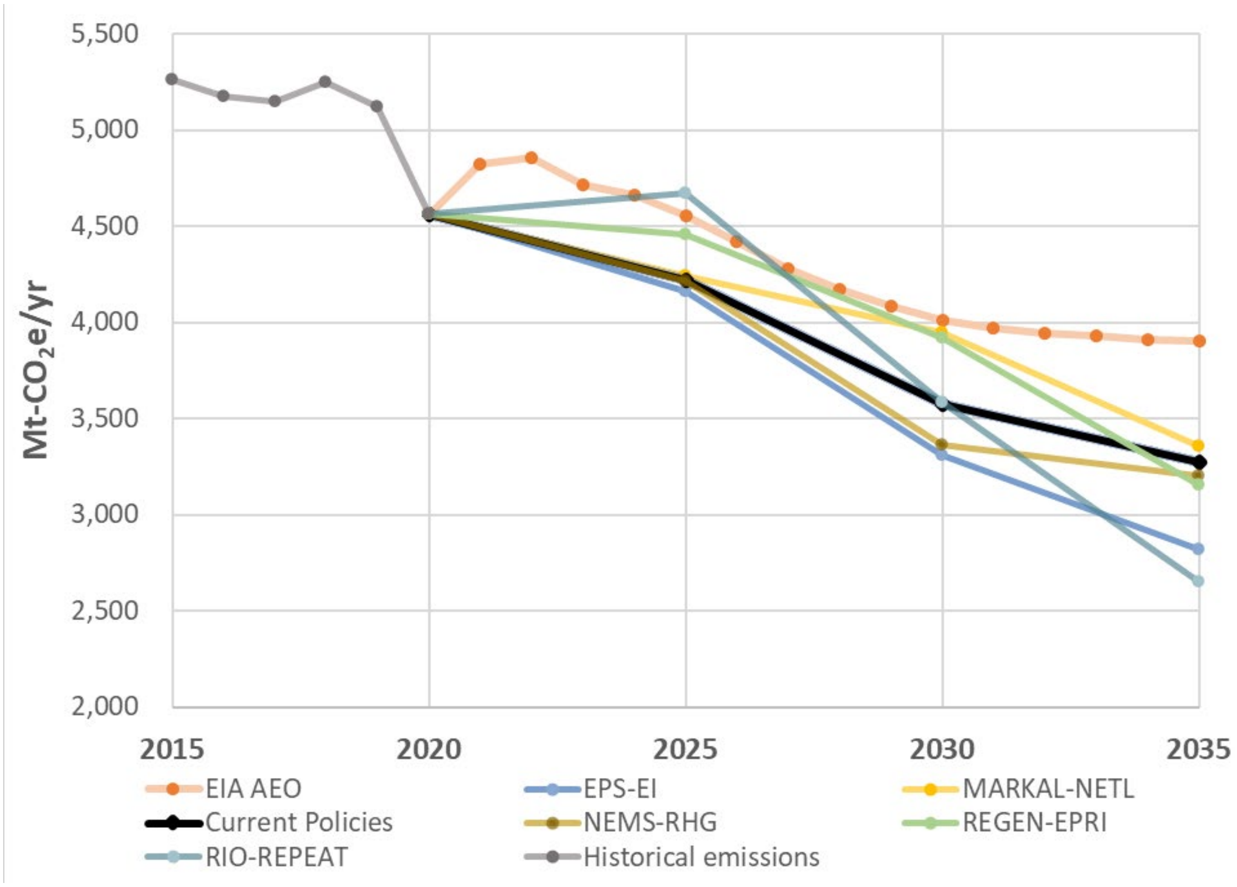

Supplementary Figure 2. Energy CO_2 emissions through 2035. Historical data from the EIA is used through 2020. This study's *Current Policies* scenario (in black) is compared against EIA AEO's baseline projection and Bistline et al.'s 2023 multi-model comparison paper on the IRA.

Supplementary Note 15. Sensitivity analysis

We also assessed emissions projections from the two scenarios by varying assumptions on a few important drivers, including GDP, population growth, oil and gas prices, solar and wind costs, and the land sink carbon sequestration potential. See Supplementary Table 8 for our sensitivity assumptions, and Supplementary Note 7 for the sources for our core assumptions.

Supplementary Table 8. Assumptions under sensitivity scenarios

Driver	Core Assumptions	Sensitivities
**GDP**	GDP is assumed to grow by 2.1% per year on average from 2020 through 2035.	**High**: GDP is assumed to grow by 3.2% per year on average through 2035.<br>**Low:** GDP grows by 1% per year on average through 2035.
**Population**	Population is assumed to grow by 0.61% per year on average from 2020 through 2035.	**High**: Grows by 0.73% per year on average through 2035.<br>**Low:** Grows by 0.48% per year on average through 2035.
**Fuel prices**	Gas prices are assumed to decrease at an average rate of 7.1% per year from 2021 through 2025. Prices increase 0.1% on average between 2025 and 2035.	**High:** Gas prices are assumed to increase at an average rate of 1.1% per year from 2021 through 2025, and continue to increase at 3.2% per year on average between 2025 and 2035.<br><br>**Low:** Gas prices are assumed to decrease at an average rate of 12.9% per year from 2021 through 2025, then increase at 2.1% per year on average between 2025 and 2035.
	Oil prices are assumed to increase at an average rate of 0.7% per year between 2021 and 2035.	**High:** Oil prices are assumed to increase at an average rate of 2.1% per year from 2021 through 2035.<br><br>**Low:** Oil prices are assumed to decrease at an average rate of 3.7% per year from 2021 through 2035.
**Solar power**	Utility solar PV capital costs are assumed to decrease by 35% from 2022 to 2035.	**High:** Utility solar PV capital costs are assumed to decrease by 47% from 2022 to 2035.<br>**Low:**Utility solar PV capital costs are assumed to decrease by 10% from 2021 to 2035.
**Wind power**	Land-based wind and offshore wind capital costs are assumed to decrease by 34% and 20%, respectively, from 2022 to 2035.	**High:** Land-based wind and offshore wind capital costs are assumed to decrease by 50% and 23%, respectively, from 2022 to 2035.<br>**Low:** Land-based wind and offshore wind capital costs are assumed to decrease by 29% and 13%, respectively, from 2022 to 2035.

**LULUCF**	LULUCF sector is assumed to sequester 926 $MtCO_2$ and 858 $MtCO_2$ by 2035 under *Enhanced Ambition* and Current *Policies*, respectively.	**High:** LULUCF sector is assumed to sequester 926 $MtCO_2$ sequestration by 2035.<br>**Low:** LULUCF sector is assumed to sequester 762 $MtCO_2$ sequestration by 2035.

Supplementary Note 16. Bottom-up aggregation analysis

Our approach to assess bottom-up actions is built upon a previously developed methodology. For a more detailed description of the aggregation and overall modeling methodology, please see the Accelerating America's Pledge Technical Appendix (2019)[1] and Hultman, et al. (2020)[2] along with its accompanying supplementary information. In this analysis, we focus on state-level action, with the assumption that additional policies from cities and businesses will also be needed to achieve enhanced state-level action.

The scope of non-federal climate actions considered in this analysis is not limited to explicit climate policies. Rather, it is inclusive of actions taken to induce cost savings, promote economic growth, and deliver health impacts, among others, but which all have the potential to drive greenhouse gas emissions reductions in the United States. The goal of the bottom-up aggregation analysis is to estimate the overall economy-wide emissions reductions resulting from a combination of climate actions by a wide range of actors.

The process involves collecting climate actions across sectors at the state level. To accomplish this, we collected data to quantify the relative impact of a set of high-impact non-federal climate actions against a no-policy case, taking care to avoid any double counting of potential emissions reductions. For example, when examining energy efficiency measures, we made sure to account for efficiency improvements already embedded in projected years. The resulting state-level impacts of these non-federal climate actions were then represented in GCAM-USA-CGS to estimate potential emissions reductions across the entire economy.

Renewable portfolio standards. We assessed legally binding state-level renewable portfolio standards (RPS), differentiating between policies that include hydroelectric and non-hydroelectric sources, in 29 U.S. states and the District of Columbia. We assume all future renewable energy demand driven by the goals is satisfied by non-hydroelectric generation.

As a first step, we collected state-level electricity load forecasts and effective RPS demand rates (representing the actual percentage of a state's electricity load needed to meet RPS

requirements for a specific year) derived from Lawrence Berkeley Lab to estimate the renewable electricity generation driven by RPS legislation.[46] We then projected state-level electricity load by coupling historical state-level electricity sales data from the EIA[47] with the annual growth rates sourced from GCAM-USA-CGS's state-level electricity demand outputs.

We assumed that all legally binding state-level RPS would be achieved in the *Current Policies* scenario. In the *Enhanced Ambition* scenario, we assumed that Tier 1 states would reach 75% renewable energy generation by 2035, which is in line with leading states such as California and New York. Tier 2 states lag but still achieve 55% renewable by 2035. Meanwhile, Tier 3 states achieve 20% renewable energy by 2035.

Coal generation. Under both scenarios, we assumed that no new coal-fired power plants without CCS would be constructed in the United States. This is consistent with the lack of new planned coal plants.[48] To represent *Current Policies*, we collected announced retirement dates for all existing coal power plants in the United States and applied these to the historical electricity generation from each of these plants in 2020 to construct a future trajectory for electricity generation from all coal power plants. These plant-level trajectories were then aggregated to the state level to produce an upper bound on electricity generation from coal in the absence of any changes in policy or economic conditions that would further accelerate the decline of coal in the power sector. The resulting state-level upper bounds on electricity generation from coal were implemented at the state level in GCAM-USA-CGS.

Announced retirement dates were collected from 3 sources: EIA-860 (2021)[49], Global Energy Monitor's Global Coal Plant Tracker (July 2022)[48], and the EPA National Electric Energy Data System (NEEDS) database (October 2022).[50] For plants in which retirement dates differ between these sources, the earliest retirement date was used. Finally, we implemented the impact of the IRA's energy community reinvestment financing program as a further reduction in total electricity generation from coal in the United States, with resultant coal plant retirements occurring in the states where economics drive faster retirements according to state-level profit-shutdown parameters.

In the *Enhanced Ambition* scenario, we assumed full phase-out of coal-fired electricity generation by 2030. Although this would be affected by a combination of bottom-up actions by utility companies, state governments, changes in consumer demand, and federal spending from the IRA, we implement this as a national constraint on coal generation reaching zero by 2030.

Nuclear. Under both scenarios, we assumed that a combination of federal and state policy actions allow for any nuclear power plants at risk of retiring to stay online through 2035. We also account for Vogtle units 3&4 in Georgia coming online and operating at full capacity by 2025 as these are the only new nuclear power stations scheduled to come online in the United States by 2035. The results of these modeling assumptions is that nuclear power generation in each model period is exogenously specified in GCAM-USA-CGS.

Zero-emission vehicle sales targets. California has become a model state for electric vehicle adoption by passing emission reduction regulations and setting ZEV sales targets. California's Advanced Clean Cars (ACC) I requires vehicle manufacturers to sell increased shares of zero-emission passenger cars and light-duty trucks starting in model year 2017 and ending in model year 2025. Under 2012 amendments, state manufacturers can opt to comply with California's enhanced standards over the EPA's GHG regulations.[51] In 2022, California passed ACC II, which extends light-duty vehicle (LDV) sales targets after 2025 with an ultimate goal of 100% sales of ZEVs by 2035.[30] Since then, a growing number of states have announced their adoption of ACC II over the less stringent EPA regulations. As states slowly adopt ACC I and ACC II regulations aimed at LDVs, there has been concurrent movement towards the electrification of medium and heavy-duty vehicles. California's Advanced Clean Trucks (ACT) program sets ZEV sale stargets for medium and heavy-duty vehicles through 2035, which has been adopted by other states as well.[31]

In *Current Policies,* we assumed that the states that have either implemented or have announced their plan to implement ACC I, ACC II and ACT achieve the sales targets. See Supplementary Table 9 and Supplementary Figure 3 below for a list of states that achieve these sales targets.

In *Enhanced Ambition*, we assumed wider adoption of California's ACC II and ACT regulations.

- For LDV electrification, Tier 1 states follow California and achieve ZEV sales targets on the same timeframe as mandated by ACC II, with ZEVs making up 25% of LDV sales by 2026, increasing to 68% in 2030, and maxing out at 100% in 2035.[30] We assumed a three-year time lag for Tier 2 states and a six year time lag for Tier 3 states for achieving ACC II targets.
- For medium and heavy-duty vehicle electrification, ACT stipulates the following ZEV sales targets between 2024 to 2035: 55% for Class 2b-3 trucks, 75% for Class 4-8

straight truck sales, and 40% for truck tractor sales. Under *Enhanced Ambition*, Tier 1 states follow California and achieve ZEV sales targets on the same timeframe as mandated by ACT. We assumed a three-year time lag for Tier 2 states and a six-year time lag for Tier 3 states for achieving ACT targets.

Across both scenarios, we took care to ensure that we were not double counting electrification in the transportation sector. We first accounted for other transportation sector policies, like CAFE, IRA tax credits for EVs, and state-level EV tax credits. If states met or exceeded their specified sales targets with these policies, we did not model additional electrification. If states did not achieve their sales targets, we modeled additional electrification that would allow them to meet the targets.

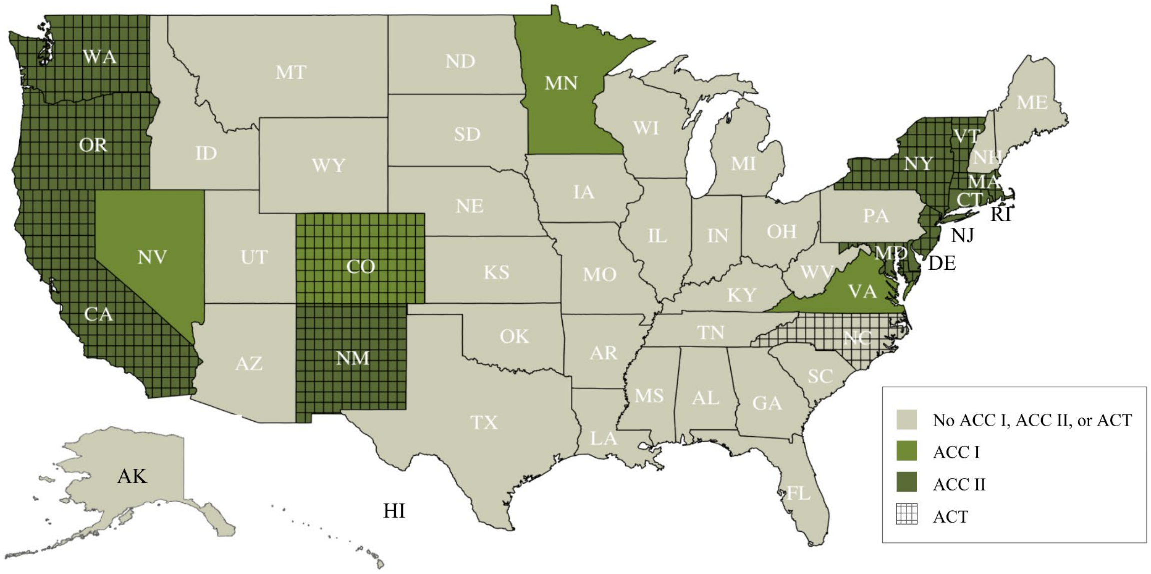

Supplementary Figure 3: Status of ACC I, ACC II, and ACT across all states.

Supplementary Table 9. States that have implemented or have announced plans to implement ACC I, ACC II, and ACT. States with an asterisk represent states that are in various stages of governor announcement and implementation of ACC II/ ACT, but have yet to make targets official.

ACC I	ACC II	ACT
Colorado	California	California
Minnesota	Connecticut *	Colorado

Nevada	Delaware *	Connecticut *
Virginia	Maryland *	Maryland *
	Massachusetts	Massachusetts
	New Jersey *	New Jersey
	New Mexico *	New Mexico *
	New York	New York
	Oregon	North Carolina *
	Rhode Island *	Rhode Island *
	Vermont	Oregon
	Washington	Vermont
		Washington

Vehicle miles traveled. Though most current climate-related transportation policies focus on vehicle emission standards and ZEV sales targets, an increasing number of states and other local actors have begun implementing vehicle miles traveled (VMT) reduction policies as a strategy for achieving GHG reduction goals. According to the U.S. Department of Transportation, VMT per capita measures " the total annual miles of vehicle travel divided by the total population in a state or in an urbanized area."[52]

Total VMT in the U.S. have been steadily increasing since the mid-20th century, while VMT per capita has been relatively constant since about 2005 (excluding the months following the outbreak of Covid-19, during which lockdowns hampered mobility).[53,54] The Federal Highway Administration (FHWA) projected a 0.6% annual average growth rate in national VMT between 2019 and 2049, with LDVs increasing at a 1.8% annual average rate and single unit truck increasing by the same average annual rate.[55] Though LDVs currently account for the majority of total VMT, FHWA projects that their growth will taper off to 0.5% annually by the 2040s. In contrast, single-unit trucks are expected to average 1.8% annual growth over the entire forecast period.[55]

California's 2022 Scoping Plan for Achieving Carbon Neutrality proposed a statewide VMT per capita reduction of 25% below 2019 levels by 2030 and 30% by 2045.[56] The scoping plan notes that this reduction target is in line with the goals of SB 375, which aims to "reduce demand for fossil transportation fuels and GHGs, and improve air quality."[56] Three

other states - Connecticut, Minnesota, and Washington - have passed legislation with varying VMT reduction targets spanning both near- and long-term policy implementation.

Since there is little precedent for VMT reduction targets, we included them only in the *Enhanced Ambition* scenario. Additionally, we assumed VMT reduction targets are applied to LDVs only. To create a baseline case, we collected 2015 LDV VMT data from the FHWA Highway Statistics Series Publications for each state and applied VMT growth rates from GCAM-USA-CGS through 2035.[57] We then created the *Enhanced Ambition* scenario by applying an annualized reduction target between 2026-2035 on top of the baseline. As an upper bound, we looked at California's current VMT target, which we estimated to have an annual per capita reduction of 1.75% between 2026 and 2030.[56] The *Enhanced Ambition* scenario includes a more conservative annualized per capita reduction rate of 1.25% for Tier 1 states, 1.00% for Tier 2 states, and 0.75% for Tier 3 states.

We implemented VMT reductions as a percentage reduction in total passenger service demand in the model. The resulting decline in passenger service was predominantly from road vehicles, but a small amount was from rail and short-haul aircraft.

Building efficiency standards. State-level Energy Efficiency Resources Standards (EERS) mandate efficiency in delivery of residential, commercial, and industrial electricity and gas by setting energy savings targets for utilities. These policies vary in scope and ambition by state; Maryland's EERS covers all sales within the state, while Arizona's only covers two large utilities, targets range from 0-2.5% of sales, and electricity standards are more common than than gas targets (data collection found 27 state-level EERS policies for electricity efficiency, and 19 policies for gas).

To calculate EERS savings under *Current Policies*, we used current state-level standards and applied them to state-level demand projections. We collected current state-level efficiency standards using ACEEE's EERS documentation,[58] which we cross-checked with individual state websites. Then, to calculate future demand projections, we used historical state-level demand by sector from the EIA for the years 1990-2019[59,60] and layered on projections of future demand obtained from GCAM-USA-CGS's state-level electricity and natural gas demand outputs through 2035.

Then, to avoid double counting, we removed embedded energy efficiency savings, or the expected efficiency gains by state in the absence of binding policies, from the calculated EERS savings. This approach allows us to implement only the additional savings that would

be achieved from EERS policies. We derived embedded energy efficiency using projections of future demand from a GCAM-USA-CGS baseline scenario along with projected energy efficiency savings reported by EIA's Annual Electric Power Industry Report.[49]

Under the *Enhanced Ambition* scenario, states begin to adopt more ambitious standards starting in 2025 (Supplementary Table 10). Tier 1 states follow New York's electricity and gas efficiency policies, which mandates annual savings of 3% and 1.3%, respectively, beginning in 2025.[61,62] Given the aggressive nature of the electricity efficiency goal, each Tier 1 state's electricity savings rate in 2020 was linearly interpolated to 3% in 2030, allowing states time to gradually reach higher efficiency levels. For Tier 2 states, we implemented electricity efficiency gains of 1.2% annually, based upon an extended version of Virginia's policy.[62] We used annual natural gas savings of 0.75%, following efficiency policies currently enacted in Michigan.[63] We assumed that Tier 3 states implement no additional efficiency targets. If the *Current Policies* EERS savings in a state is more ambitious than what is assumed under the *Enhanced Ambition* tiering, we maintained the former savings levels. For states lacking an efficiency standard, we enacted the corresponding *Enhanced Ambition* target at 50% ambition in 2025, rising to 100% of the goal in 2030. For example, North Carolina, a Tier 2 state, has no gas EERS policy, so under the *Enhanced Ambition* scenario, we applied a gas target of 0.38% from 2025-2029, and savings of 0.75% from 2030 onwards.

Supplementary Table 10. Implementation of EERS in the *Enhanced Ambition* scenario

	Tier 1	Tier 2	Tier 3
Electricity	Linearly interpolate between 2020 savings rate and 2030 goal of 3% annual savings.	1.2% annual savings beginning in 2025<br><br>If no existing EERS:<br>0.6% beginning in 2025,<br>1.2% beginning in 2030	n/a
Natural Gas	1.3% beginning in 2025<br><br>If no existing EERS:<br>0.65% beginning in 2025,<br>1.30% beginning in 2030	0.75% beginning in 2025<br><br>If no existing EERS:<br>0.38% beginning in 2025,<br>0.75% beginning in 2030	n/a
For both technologies and all Tiers, if the *Current Policies* savings target is higher than the *Enhanced Ambition* target, maintain the *Current Policies* target until surpassed by *Enhanced Ambition* in ambition			

Building electrification standards. Though not as widespread as efficiency standards, states throughout the country have enacted policies banning use of combustion-powered appliances or otherwise encouraging electrification. Appliance bans in California[64], DC[65],

New York[66], and Washington[67] prohibit installation of fossil-fuel hookups in new construction or, in California beginning in 2030, all new fossil appliance sales. California's ban includes fossil-fuel powered space heaters and water heaters, while New York's and DC's policies mandate all-of-building electrification. Other state policies include electrification targets (e.g. New Jersey)[68], mandated reductions in emissions or energy use in the building sector (e.g. Maryland and Minnesota)[69,70], and electric-ready hookup requirements (e.g. Colorado and Delaware).[71,72] Electrification mandates, particularly for cookstoves, have encountered fierce opposition: 24 states have laws that prohibit cities from banning natural gas.[73] However, a large majority of the states with these preemptive bans are listed as Tier 3 in our analysis, and not predicted to enact ambitious electrification policies.

Building electrification under *Enhanced Ambition* is based on California's zero-emissions appliance standards, which requires that all new sales of space heaters and gas heaters comply with a zero-NO_x standard beginning in 2030.[64] We assumed that Tier 1 states follow California and pass similarly ambitious appliance turnover mandates that result in 100% electric sales for hot water and space heaters by 2030. Tier 2 states achieve this target by 2035. No enhanced ambition is assumed for Tier 3 states.

Supplementary Note 17. Land Use, Land-use Change, and Forestry

For the land use, land-use change, and forestry (LULUCF) sector, projected CH_4 emissions are estimated based on the EPA's MAC curves, as described in Supplementary Table 6. CO_2 and N_2O emissions are estimated based on results from the *America Is All In* lands sector report as described in Supplementary Table 6.[41] This report uses the Forest and Agricultural Sector Optimization Model with Greenhouse Gases (FASOMGHG) to assesses the cost-effective combination of different land-based activities to maximize the greenhouse gas mitigation potential of current federal and non-federal policies.[74] The model accounts for opportunity costs as agriculture and forestry commodity markets adjust in response to GHG reduction investments, and projects subnational land sector dynamics for 11 regions in the United States.

This report examines three scenarios under alternative policy assumptions: A baseline, no climate action scenario, an Existing Policies scenario, and an Enhanced Ambition scenario. The Existing Policies scenario assumes full implementation of current federal and state policies, including $42 billion in investments in climate-smart agricultural practices, conservation policies across the forestry and agriculture sectors, wildfire mitigation, and afforestation and reforestation efforts. The Enhanced Ambition scenario assumes

heightened ambition from climate-leading, fast-mover states, leading to an increase of $160 billion in climate-smart policies and incentives for the agriculture and forestry sectors.

The agriculture and forestry emissions projections resulting from these investments under the Existing Policies and Enhanced Ambition scenarios are used in this paper's *Current Policies* and *Enhanced Ambition* scenarios, respectively.

Supplementary Figures

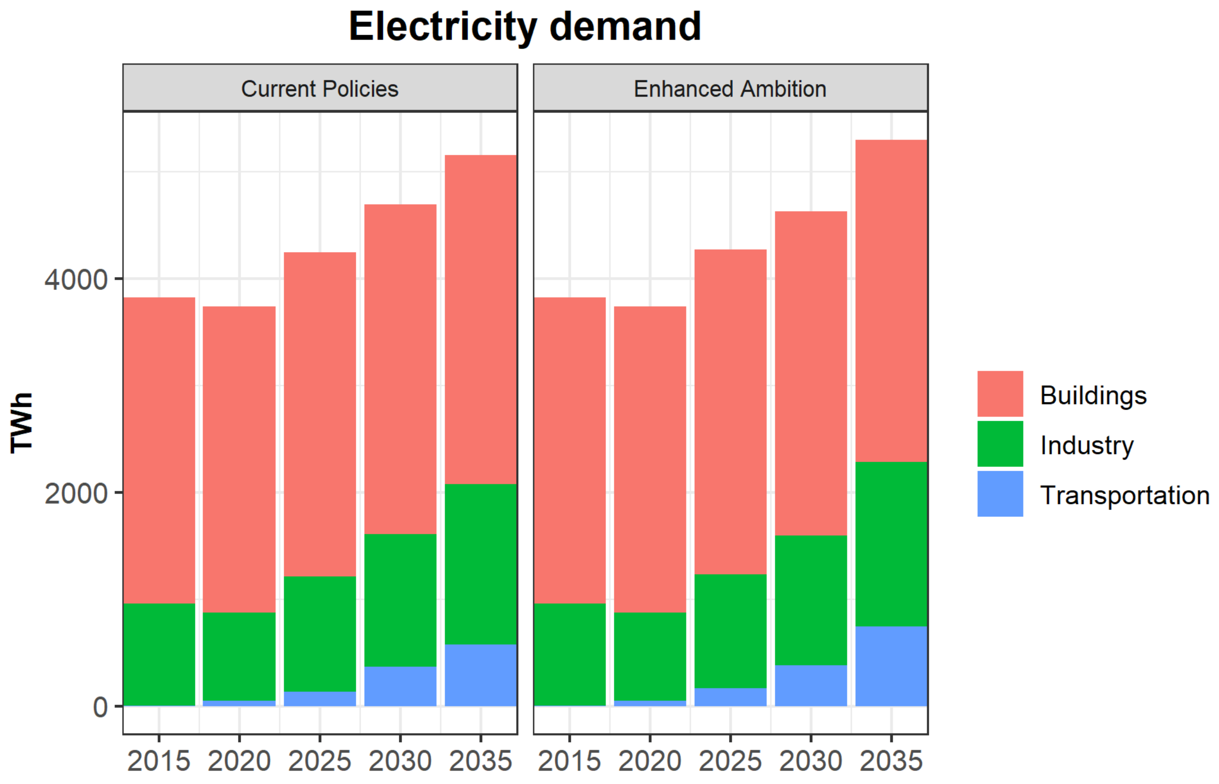

Supplementary Figure 4. Electricity demanded by the buildings, industry and transportation sectors in the *Current Policies* and *Enhanced Ambition* scenarios, in units of TWh. Electricity demand does not increase dramatically in the Enhanced Ambition scenario due to additional efficiency measures that decrease total demand in the scenario.

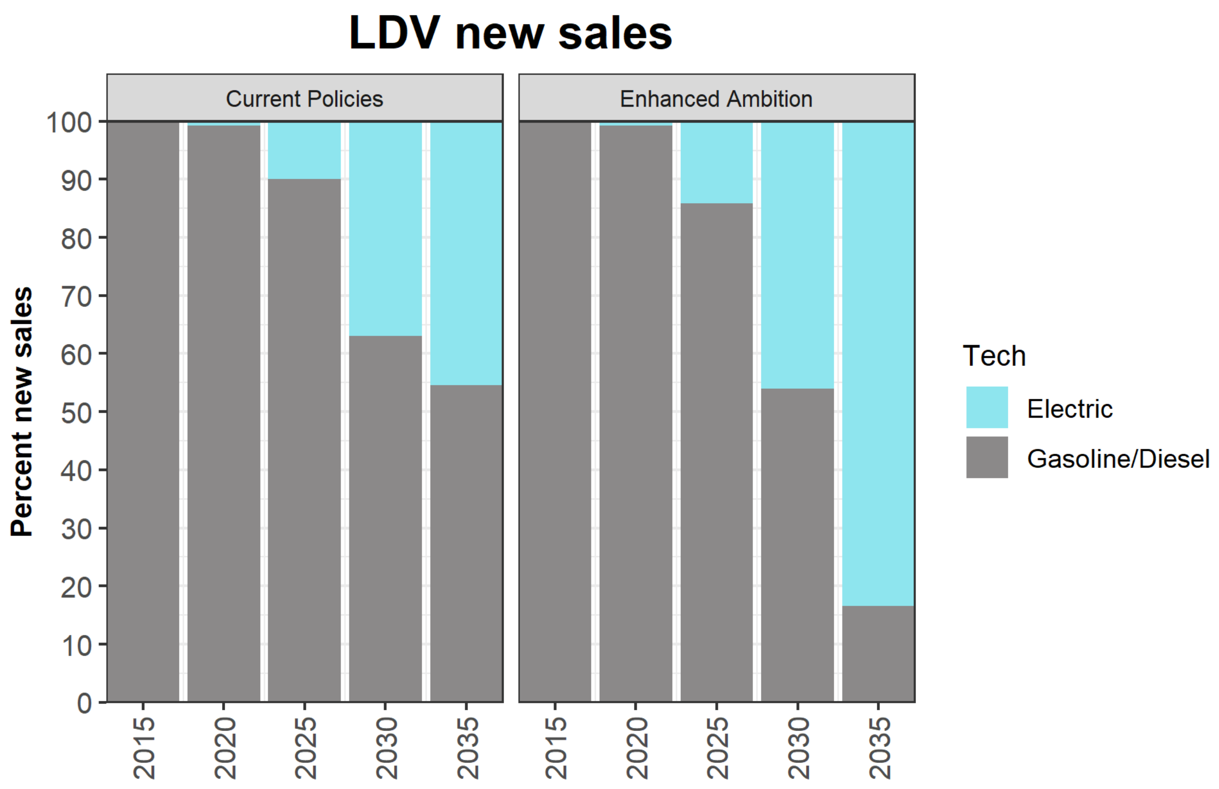

Supplementary Figure 5. LDV new sales in the *Current Policies* and *Enhanced Ambition* scenarios, in units of percentages. The percentage of new sales from electric vehicles is in blue, and the percentage from gasoline/diesel is in gray. Under the *Enhanced Ambition* scenario, the share of EV sales increases to 83% in 2035 as a result of additional EV incentives and mandates, compared to 45% under the *Current Policies scenario.*

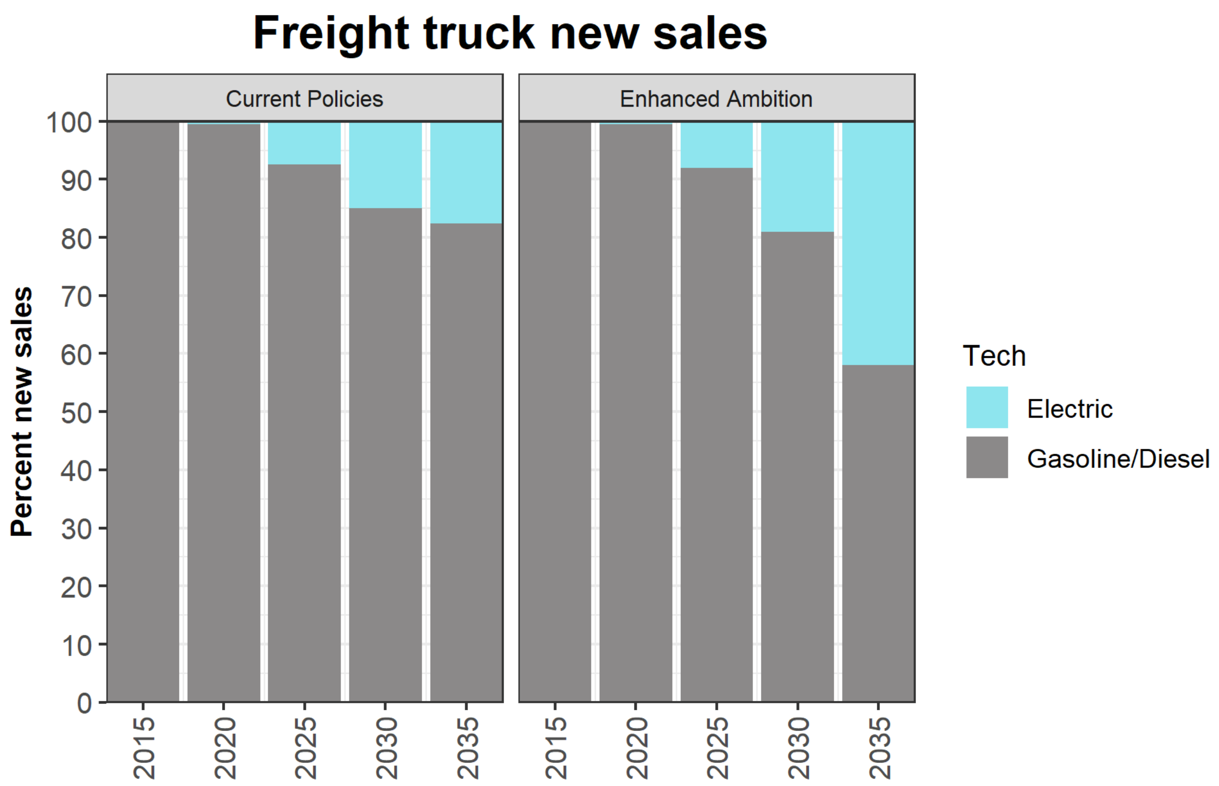

Supplementary Figure 6. Freight truck new sales in the *Current Policies* and *Enhanced Ambition* scenarios, in units of percentages. The percentage from electric vehicles is in blue, and the percentage from gasoline/diesel service is in gray. Under the *Enhanced Ambition* scenario, electrification increases to 42% as a result of additional EV incentives and mandates, compared to 18% under the *Current Policies* scenario.

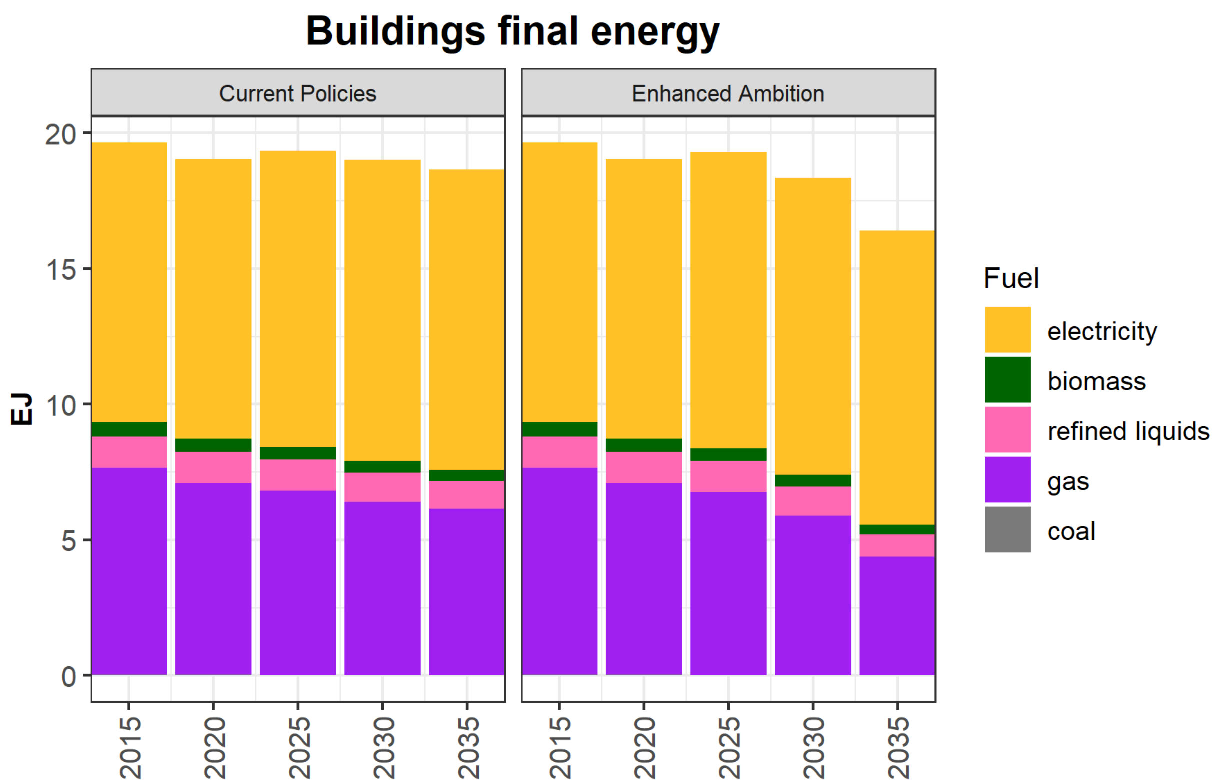

Supplementary Figure 7. Buildings sector final energy demand in the *Current Policies* and *Enhanced Ambition* scenarios, in units of exajoules (EJ). Under the *Enhanced Ambition* scenario, the share of electricity increases from 54% in 2020 to 66% by 2035 as a result of electrification incentives and mandates, compared to 59% under the *Current Policies* scenario. Additionally, total demand decreases in the *Enhanced Ambition* scenario as a result of enhanced energy efficiency measures as well the higher share of electricity, which is more energy efficient than other fuels.

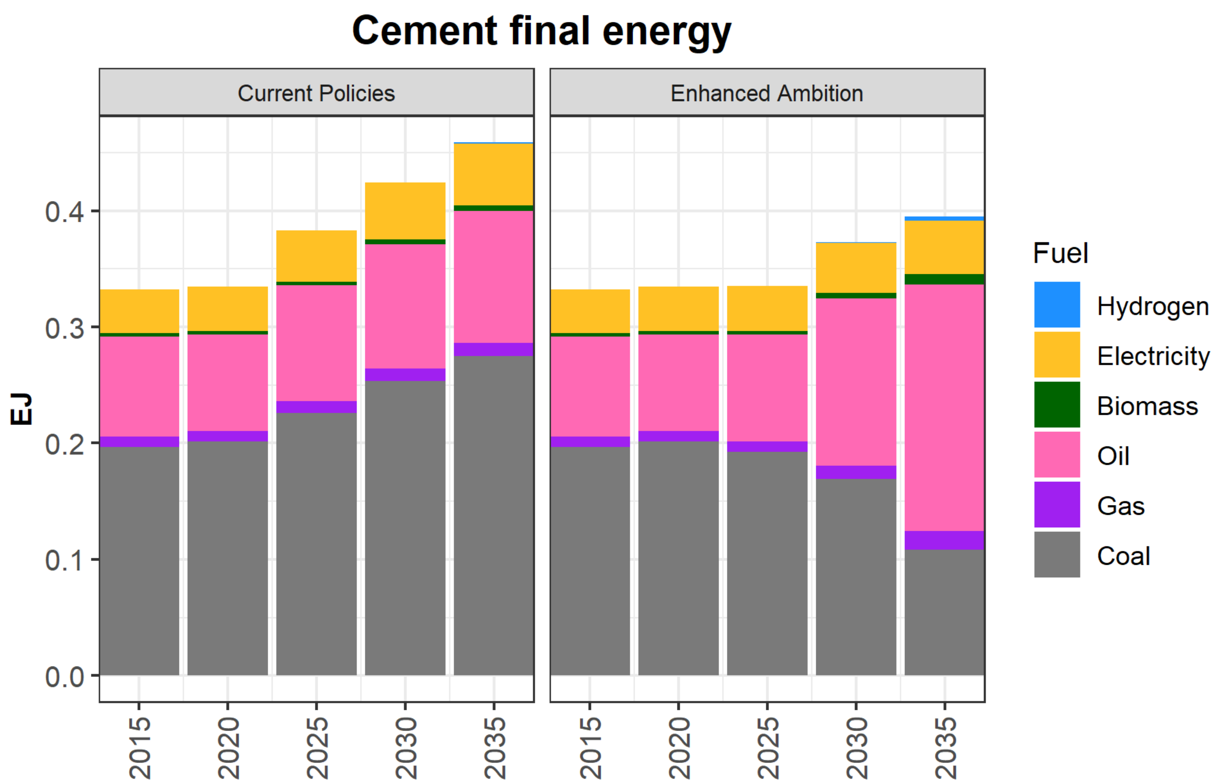

Supplementary Figure 8. Cement sector final energy demand in the *Current Policies* and *Enhanced Ambition* scenarios, in units of exajoules (EJ).

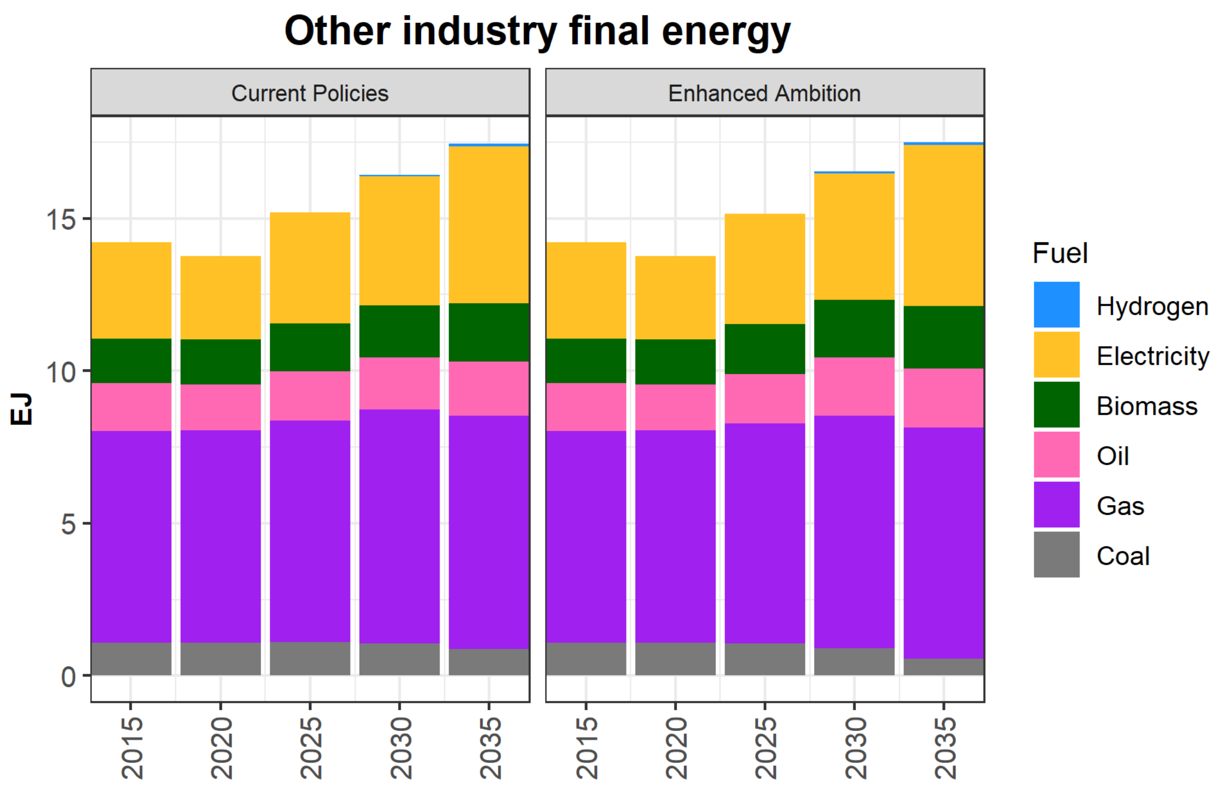

Supplementary Figure 9. Other industrial sector final energy in the *Current Policies* and *Enhanced Ambition* scenarios, in units of exajoules (EJ).

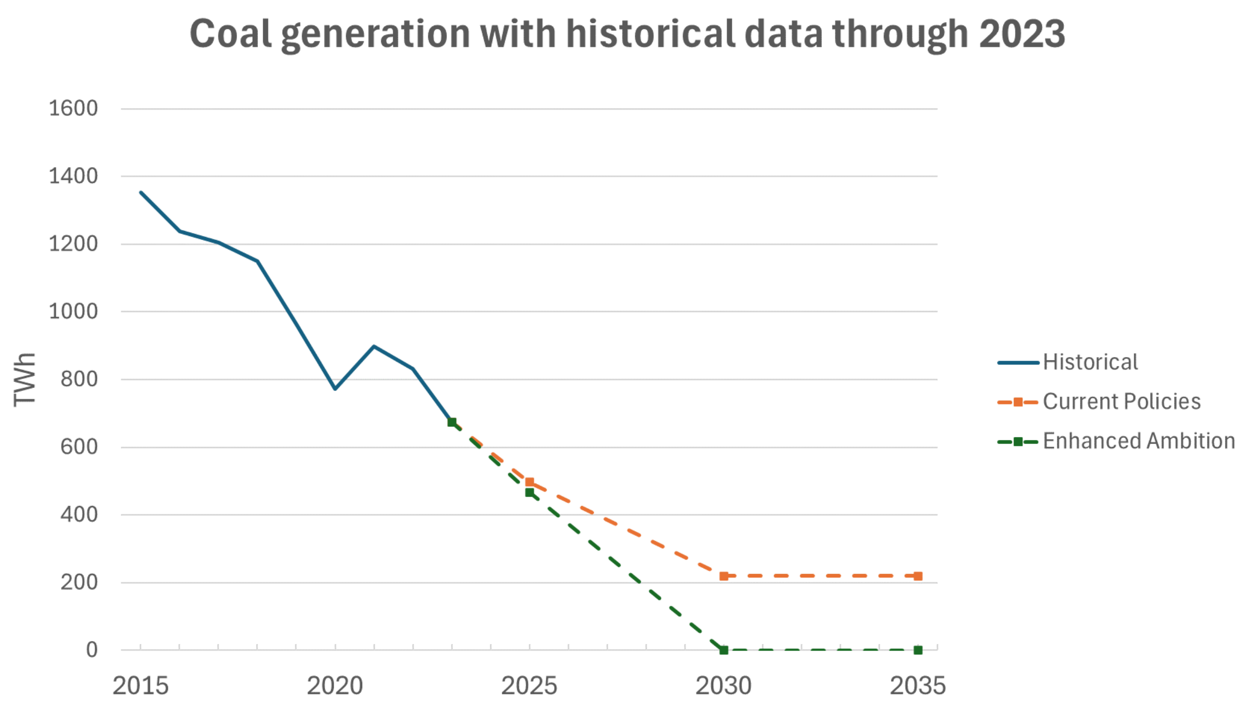

Supplementary Figure 10. Coal generation, with historical data through 2023 from the U.S. Energy Information Administration, and projections under the Current Policies and Enhanced Ambition scenarios, in units of TWh. Markers indicate the model years; values for the years between model years are estimated using a linear interpolation.

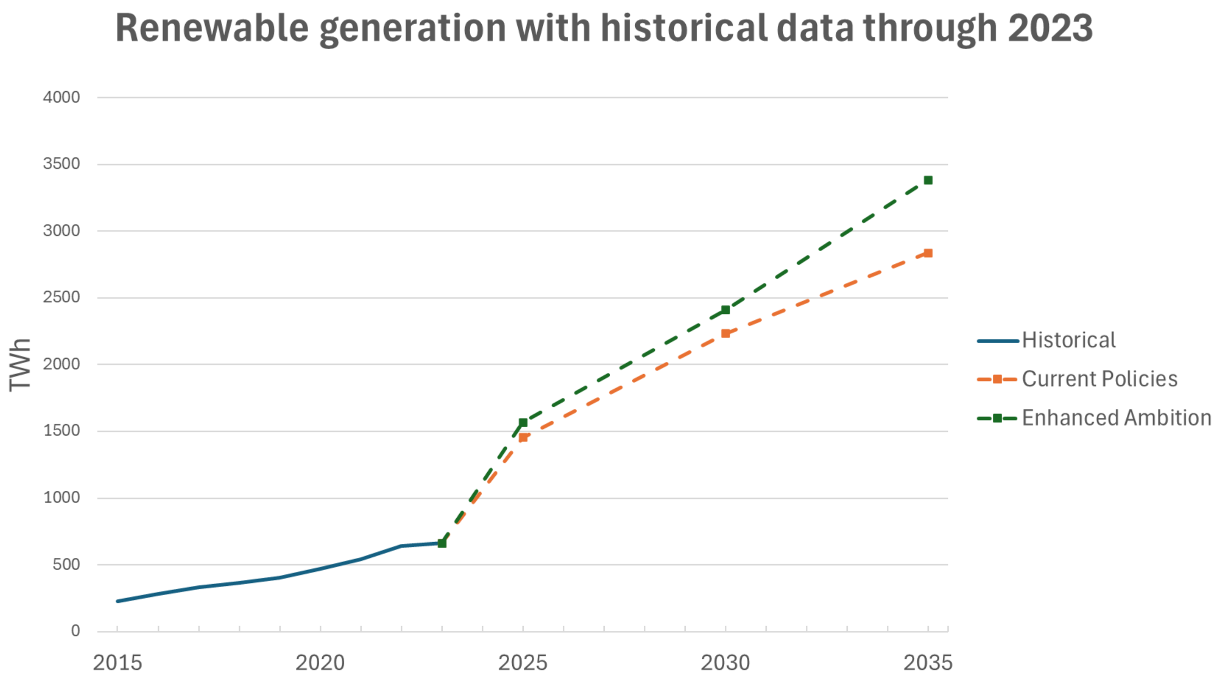

Supplementary Figure 11. Renewable generation (solar and wind), with historical data through 2023 from the U.S. Energy Information Administration, and projections under the Current Policies and Enhanced Ambition scenarios, in units of TWh. Markers indicate the model years; values for the years between model years are estimated using a linear interpolation.

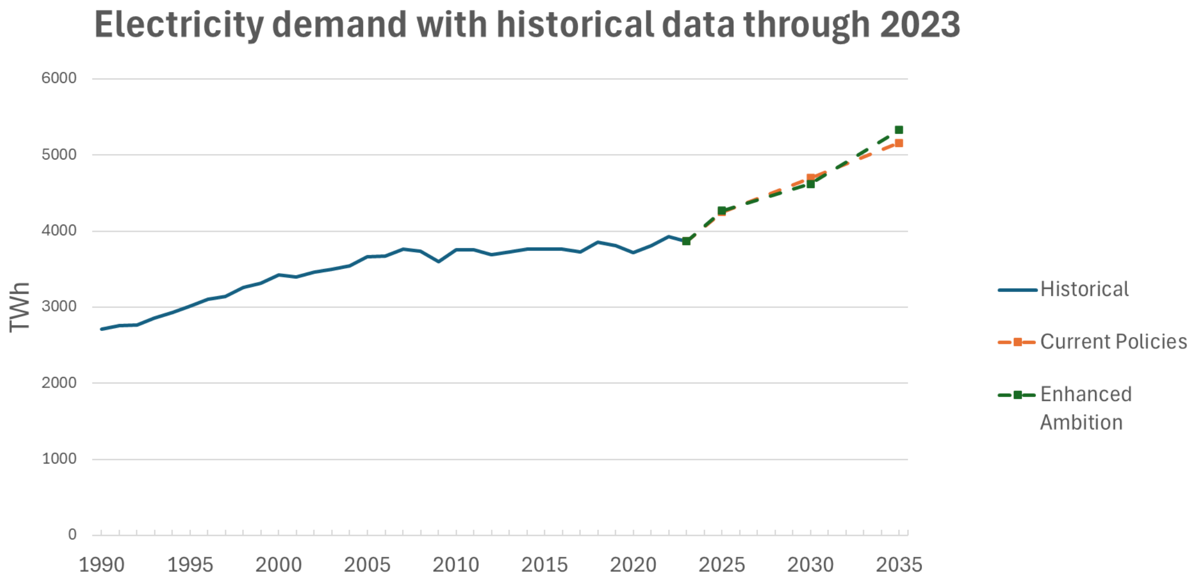

Supplementary Figure 12. Electricity demand, with historical data through 2023 from the U.S. Energy Information Administration, and projections under the Current Policies and Enhanced Ambition scenarios, in units of TWh. Markers indicate the model years; values for the years between model years are estimated using a linear interpolation.

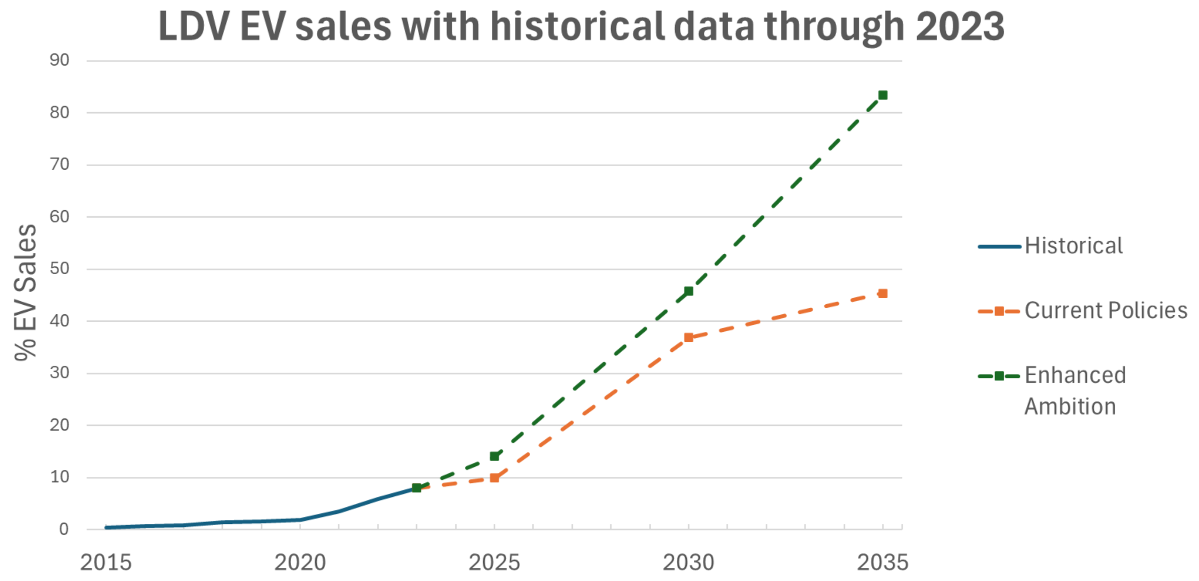

Supplementary Figure 13. Light-duty vehicle EV sales, with historical data through 2023 from the Transportation Energy Data Book and Argonne National Laboratory and projections under the Current Policies and Enhanced Ambition scenarios. Markers indicate the model years; values for the years between model years are estimated using a linear interpolation. EVs include all-electric vehicles and plug-in hybrid electric vehicles, assuming an average utility factor of 37%.

Supplementary References

1. Hultman, N. *et al. Accelerating America's Pledge: Technical Appendix. The America's Pledge Initiative on Climate Change.* 62 (2019).
2. Hultman, N. E. *et al.* Fusing subnational with national climate action is central to decarbonization: the case of the United States. *Nat. Commun.* **11**, 5255 (2020).
3. Kennedy, K. *et al. Blueprint 2030: An All-In Climate Strategy for Faster, More Durable Emissions Reductions*. https://www.americaisallin.com/blueprint-2030 (2022).
4. Hultman, N. *et al. An All-In Climate Strategy Can Cut U.S. Emissions by 50% by 2030*. https://cgs.umd.edu/research-impact/publications/all-climate-strategy-can-cut-us-emissions-50-2030 (2021).
5. Zhao, A. *et al. An "All-In" Pathway To 2030: U.S. Methane Emissions Reduction Potential*. https://cgs.umd.edu/research-impact/publications/all-pathway-2030-us-methane-emissions-reduction-potential (2022).
6. Yarmuth, Rep. J. A. *Inflation Reduction Act of 2022*. (2022).
7. Federal Transit Administration. Bipartisan Infrastructure Law. *US Department of Transportation* https://www.transit.dot.gov/BIL (2023).
8. US Environmental Protection Agency. *EPA Issues Supplemental Proposal to Reduce Methane and Other Harmful Pollution from Oil and Natural Gas Operations*. https://www.epa.gov/controlling-air-pollution-oil-and-natural-gas-industry/epa-issues-supplemental-proposal-reduce (2022).
9. US Environmental Protection Agency. *EPA Proposes New Carbon Pollution Standards for Fossil Fuel-Fired Power Plants to Tackle the Climate Crisis and Protect Public Health*. https://www.epa.gov/newsreleases/epa-proposes-new-carbon-pollution-standards-fossil-fuel-fired-power-plants-tackle (2023).
10. Massachusetts Executive Office of Energy and Environmental Affairs. *MA*

Decarbonization Roadmap. https://www.mass.gov/info-details/ma-decarbonization-roadmap (2020).

11. City of San Diego. *Climate Action Plan*. https://www.sandiego.gov/sustainability/climate-action-plan (2022).
12. Maryland Public Service Commission. Renewable Energy. *Maryland Public Service Commission* https://www.psc.state.md.us/electricity/renewable-energy/ (2023).
13. Austin Energy. Renewable Power Generation. https://austinenergy.com/about/environment/renewable-power-generation (2022).
14. Bay Area Air Quality Management District. Air District strengthens building appliance rules to reduce harmful NOx emissions, protect air quality and public health. *Bay Area Air Quality Management District* https://www.baaqmd.gov/news-and-events/page-resources/2023-news/031523-ba-rules (2023).
15. North Carolina Department of Environmental Quality. Advanced Clean Trucks. *North Carolina Department of Environmental Quality* https://www.deq.nc.gov/about/divisions/air-quality/motor-vehicles-and-air-quality/advanced-clean-trucks.
16. Dwyer, K. 'We are full': IndyGo seeking to borrow $65 million for new buses and garage. *The Indianapolis Star* https://www.indystar.com/story/news/local/transportation/2021/11/02/indygo-seeks-65-million-loan-purple-line-buses-new-bus-garage-indianapolis-marion-county-indiana/6133660001/ (2021).
17. Maez, M. New Mexico adopts nationally leading oil and gas emissions rule. *New Mexico Environment Department* https://www.env.nm.gov/wp-content/uploads/2022/04/2022-04-14-COMMS-New-Mexico-adopts-nationally-leading-oil-and-gas-emissions-rule-Final.pdf (2022).
18. Bond-Lamberty, B. *et al.* GCAM 6.0. Joint Global Change Research Institute https://doi.org/10.5281/ZENODO.6619287 (2022).
19. National Renewable Energy Laboratory. 2022 Annual Technology Baseline.

https://atb.nrel.gov/electricity/2022/index (2022).

20. US Environmental Protection Agency. Global Non-CO2 Greenhouse Gas Emission Projections & Mitigation Potential: 2015-2050. https://www.epa.gov/global-mitigation-non-co2-greenhouse-gases/global-non-co2-greenhouse-gas-emission-projections (2019).

21. Kennedy, K. *et al. Blueprint 2030: An All-In Climate Strategy for Faster, More Durable Emissions Reductions*. https://www.americaisallin.com/blueprint-2030 (2022).

22. Zhao, A. *et al. An All-In Pathway to 2030: The Beyond 50 Scenario*. https://www.americaisallin.com/Beyond50 (2022).

23. Annual Energy Outlook 2023 - U.S. Energy Information Administration (EIA). https://www.eia.gov/outlooks/aeo/index.php.

24. Larsen, K. *et al. Taking Stock 2020: The COVID-19 Edition*. 18 https://rhg.com/research/taking-stock-2020/ (2020).

25. *Inflation Reduction Act of 2022*. *H.R.5376*.

26. US EPA, O. Greenhouse Gas Standards and Guidelines for Fossil Fuel-Fired Power Plants. https://www.epa.gov/stationary-sources-air-pollution/greenhouse-gas-standards-and-guidelines-fossil-fuel-fired-power (2023).

27. The Regional Greenhouse Gas Initiative (RGGI). https://www.rggi.org/.

28. Corporate Average Fuel Economy. *National Highway Traffic Safety Administration* https://www.nhtsa.gov/laws-regulations/corporate-average-fuel-economy (2022).

29. US EPA, O. Final Rule and Related Materials for Control of Air Pollution from New Motor Vehicles: Heavy-Duty Engine and Vehicle Standards. https://www.epa.gov/regulations-emissions-vehicles-and-engines/final-rule-and-related-materials-control-air-pollution (2022).

30. California Air Resources Board. Advanced Clean Cars II. https://ww2.arb.ca.gov/our-work/programs/advanced-clean-cars-program/advanced-clean-cars-ii.

31. California Air Resources Board. Advanced Clean Trucks Fact Sheet. *ca.gov* https://ww2.arb.ca.gov/resources/fact-sheets/advanced-clean-trucks-fact-sheet (2021).

32. *Statewide Multimodal Tranportation Plan*. https://www.minnesotago.org/application/files/4216/7165/1126/2022_SMTP_ExecutiveSummary_12202022.pdf (2022).

33. California Air Resources Board. 2022 Scoping Plan for Achieving Carbon Neutrality. (2022).

34. US Energy Information Administration. Annual Energy Outlook 2023. https://www.eia.gov/outlooks/aeo/tables_ref.php (2023).

35. Josh Becker. *Greenhouse Gases: Cement Sector: Net-Zero Emissions Strategy. SB-596*.

36. Larsen, J., King, B., Hiltbrand, G. & Jones, W. Capturing the Moment: Carbon Capture in the American Jobs Plan. *Rhodium Group* https://rhg.com/research/carbon-capture-american-jobs-plan/ (2021).

37. Kuparinen, K., Vakkilainen, E. & Tynjälä, T. Biomass-based carbon capture and utilization in kraft pulpmills. *Mitig. Adapt. Strateg. Glob. Change* **24**, 1213–1230 (2019).

38. Rissman, J. *Decarbonizing Low-Temperature Industrial Heat in the U.S.* https://energyinnovation.org/wp-content/uploads/2022/10/Decarbonizing-Low-Temperature-Industrial-Heat-In-The-U.S.-Report-1.pdf (2022).

39. Jenkins, J. D., Farbes, J., Jones, R. & Mayfield, E. REPEAT Project Section-by-Section Summary of Energy and Climate Policies in the 117th Congress. https://doi.org/doi: 10.5281/zenodo.6993118 (2022).

40. Zhao, A. *et al. An "All-In" Pathway To 2030: U.S. Methane Emissions Reduction Potential*. https://cgs.umd.edu/research-impact/publications/all-pathway-2030-us-methane-emissions-reduction-potential (2022).

41. Kennedy, S. *et al. Harnessing the Land Sector to Achieve U.S. Climate Goals: An All-of-Society Approach to Meeting Our Climate Goals and Bolstering the Carbon Sink by 2035*. https://cgs.umd.edu/sites/default/files/2024-01/file_All-In%20Lands%20Report_Final.pdf

(2024).

42. Guel, T. *et al. Direct Air Capture: A Key Technology for Net Zero*. https://iea.blob.core.windows.net/assets/78633715-15c0-44e1-81df-41123c556d57/DirectAirCapture_Akeytechnologyfornetzero.pdf (2022).

43. US Environmental Protection Agency. Inventory of U.S. Greenhouse Gas Emissions and Sinks: 1990-2021. *Greenhouse Gas Emissions* https://www.epa.gov/ghgemissions/inventory-us-greenhouse-gas-emissions-and-sinks-1990-2021 (2023).

44. Stocker, T. F. *et al. Climate Change 2013: The Physical Science Basis. Contribution of Working Group I to the Fifth Assessment Report of the Intergovernmental Panel on Climate Change*. 1535 https://www.ipcc.ch/report/ar5/wg1/ (2013).

45. Bistline, J. *et al.* Emissions and energy impacts of the Inflation Reduction Act. *Science* **380**, 1324–1327 (2023).

46. Lawrence Berkeley National Lab. RPS and CES Demand Projections. (2023).

47. US Energy Information Administration. EIA-861 Annual Electric Power Industry Report 2010–2022. *Historical State Data* https://www.eia.gov/electricity/data/state/ (2022).

48. Global Energy Monitor. Global Coal Plant Tracker. *Global Energy Monitor* https://globalenergymonitor.org/projects/global-coal-plant-tracker/ (2022).

49. US Energy Information Administration. EIA-860A/860B. (2023).

50. US Environmental Protection Agency. National Electric Energy Data System (NEEDS). *Power Sector Modeling* https://www.epa.gov/power-sector-modeling/national-electric-energy-data-system-needs (2022).

51. US Department of Energy. Zero Emission Vehicle (ZEV) Production Requirements. *Alternative Fuels Data Center* https://afdc.energy.gov/laws/4249.

52. US Department of Transportation. VMT Per Capita. *transportation.gov* https://www.transportation.gov/mission/health/vmt-capita (2015).

53. FRED. Moving 12-Month Total Vehicle Miles Traveled. *Federal Reserve Bank of St.*

Louis https://fred.stlouisfed.org/series/M12MTVUSM227NFWA (2023).

54. Davis, S. C. & Boundy, R. G. *Transportation Energy Data Book Edition 40*. https://tedb.ornl.gov/wp-content/uploads/2022/03/TEDB_Ed_40.pdf (2022).

55. Office of Highway Policy Information Federal Highway Administration. FHWA Forecasts of Vehicle Miles Traveled (VMT): Spring 2023. (2023).

56. California Air Resources Board. *2022 Scoping Plan for Achieving Carbon Neutrality*. 299 https://ww2.arb.ca.gov/sites/default/files/2023-04/2022-sp.pdf (2022).

57. Federal Highway Administration. Highway Statistics Series. (2022).

58. American Council for an Energy-Efficient Economy. Energy Efficiency Resource Standards. https://database.aceee.org/state/energy-efficiency-resource-standards.

59. US Energy Information Administration. Natural Gas Delivered to Consumers. https://www.eia.gov/dnav/ng/ng_cons_sum_a_EPG0_vgt_mmcf_m.htm (2023).

60. US Energy Information Administration. Annual Electric Power Industry Report, Form EIA-861 detailed data files. https://www.eia.gov/electricity/data/eia861/ (2023).

61. NYSERDA. *New Efficiency: New York*. https://www.nyserda.ny.gov/About/Publications/New-Efficiency (2018).

62. American Council for an Energy-Efficient Economy. Energy Efficiency Resource Standards. https://database.aceee.org/state/energy-efficiency-resource-standards.

63. Michigan Public Service Commission. Energy Waste Reduction. https://www.michigan.gov/mpsc/consumer/ewr.

64. California Air Resources Board. 2022 State Strategy for the State Implementation Plan. (2022).

65. Councilmember Robert C. White, Jr. *Greener Government Buildings Amendment Act of 2022. Bill 24-785* vols A24-0755 (2023).

66. State of New York. *Enacted Budget Financial Plan*. https://www.budget.ny.gov/pubs/archive/fy24/en/fy24en-fp.pdf (2023).

67. Bauer, L. & Braff, P. Building Electrification. *Engage Olympia* https://engage.olympiawa.gov/building-electrification (2023).

68. Philip D. Murphy. *Target To Install Zero-Carbon-Emission Space Heating And Cooling Systems In 400,000 Homes And 20,000 Commercial Properties And Make 10% Of All Low-To-Moderate Income (LMI) Properties Electrification-Ready By 2030*. *Executive Order 316* (2023).

69. Maryland Department of the Environment. Building Energy Performance Standards. *Department of the Environment* https://mde.maryland.gov/programs/air/ClimateChange/Pages/default.aspx.

70. Jossi, F. Minnesota lawmakers update commercial building code aimed at energy savings. *MinnPost* https://www.minnpost.com/environment/2023/05/minnesota-lawmakers-update-commercial-building-code-amid-flurry-of-energy-bills/ (2023).

71. Perkins, H. & Chu, J. New Colorado Building Energy Code Paves Way for More All-Electric Buildings, Widespread Use of EVs. *America Is All In* https://www.americaisallin.com/new-colorado-building-energy-code-paves-way-more-all-electric-buildings-widespread-use-evs (2023).

72. Delaware General Assembly. *County or Municipal Building, Plumbing, Electrical and Other Codes*. *Code for Energy Conservation.* vol. 7602.

73. Mejia Cunningham, A. & Narita, K. Gas Interests Threaten Local Authority. *National Resources Defense Council* https://www.nrdc.org/bio/alejandra-mejia-cunningham/gas-interests-threaten-local-authority (2021).

74. Darius Adams, Ralph Alig, Bruce A. McCarl, & Brian C. Murray. FASOMGHG Conceptual Structure, and Specification: Documentation. (2005).
